# Fornix as an imaging marker for episodic memory deficits in healthy aging and in various neurological disorders

**DOI:** 10.3389/fnagi.2014.00343

**Published:** 2015-01-14

**Authors:** Vanessa Douet, Linda Chang

**Affiliations:** Department of Medicine, John A. Burns School of Medicine, University of HawaiiHonolulu, HI, USA

**Keywords:** fornix, development, aging, episodic memory, neuropsychiatric disorders, DTI

## Abstract

The fornix is a part of the limbic system and constitutes the major efferent and afferent white matter tracts from the hippocampi. The underdevelopment of or injuries to the fornix are strongly associated with memory deficits. Its role in memory impairments was suggested long ago with cases of surgical forniceal transections. However, recent advances in brain imaging techniques, such as diffusion tensor imaging, have revealed that macrostructural and microstructural abnormalities of the fornix correlated highly with declarative and episodic memory performance. This structure appears to provide a robust and early imaging predictor for memory deficits not only in neurodegenerative and neuroinflammatory diseases, such as Alzheimer's disease and multiple sclerosis, but also in schizophrenia and psychiatric disorders, and during neurodevelopment and “typical” aging. The objective of the manuscript is to present a systematic review regarding published brain imaging research on the fornix, including the development of its tracts, its role in various neurological diseases, and its relationship to neurocognitive performance in human studies.

## Introduction

The fornix is part of the limbic system that comprises cortical and subcortical structures. The cortical structures include cingulate and parahippocampal gyri, as well as the entorhinal cortex. The subcortical structures comprise the amygdalae, septal nuclei, nucleus accumbens, mammillary bodies, hypothalamus, anterior nucleus of the thalami, hippocampi and fornix.

The limbic system was first described by Pierre Paul Broca [1827–1880] (Broca, 1890) and was proposed to be the circuit of emotional experience and behavior by James W. Papez [1883–1958] (Papez, 1937). Later, its functions were linked to pleasure and reward, as well as memory and integration of memories (Rajmohan and Mohandas, 2007). Episodic memory belongs to the long-term memory system, and refers to conscious recollection of specific events (episodes) and contexts (time and place). Episodic memory frequently declines with aging and often becomes deficient in neurodegenerative diseases (Samson and Barnes, 2013) and psychiatric disorders (White et al., 2008). The critical subcortical structure for memory functions is the hippocampus (Penfield and Milner, 1958). As the major efferent white matter tract from the hippocampus, the fornix was frequently evaluated in relation to hippocampi and to memory impairments, especially to deficits in episodic memory (Yanike and Ferrera, 2014). Cumulative data from structural and diffusion tensor imaging (DTI) studies suggest that forniceal measures correlate with episodic memory performance in various neuropathological conditions, as well as during “typical” brain development and brain aging. The fornix appears to be a robust imaging predictor of episodic memory performance, independent of age and the etiology that may affect the integrity of the fornix.

In this review, we will focus on the findings from imaging studies of the fornix including its development, its implication in cognitive performance, and the structural changes associated with typical aging and neurodegenerative disorders. After a brief description of its anatomy, we will summarize the studies conducted on the forniceal formation across the lifespan, particularly those assessed by DTI which provided much new knowledge in our understanding of the fornix. We will then concentrate on diseases that may lead to an impaired or underdeveloped fornix and its likely consequences on cognitive performance, particularly in episodic memory.

## Materials and methods

We searched in the PubMed® database for relevant publications during the last decade (last update on 2014 November 15th). Our search terms included “MRI,” “DTI,” diseases of interest, “aging,” “development,” “cognition,” “memory” in combination with “fornix.” 482 results were obtained. We screened the abstracts and included only the papers that were original, published in English and referred to human research. However, the most important selection criterion was that the studies explicitly reported imaging findings of the fornix. Conference abstracts and case reports were excluded. After screening all relevant studies and excluding those papers not fulfilling the inclusion criteria, we evaluated 143 studies in further detail.

### Anatomy of the fornix (Figure 1)

**Figure 1:**
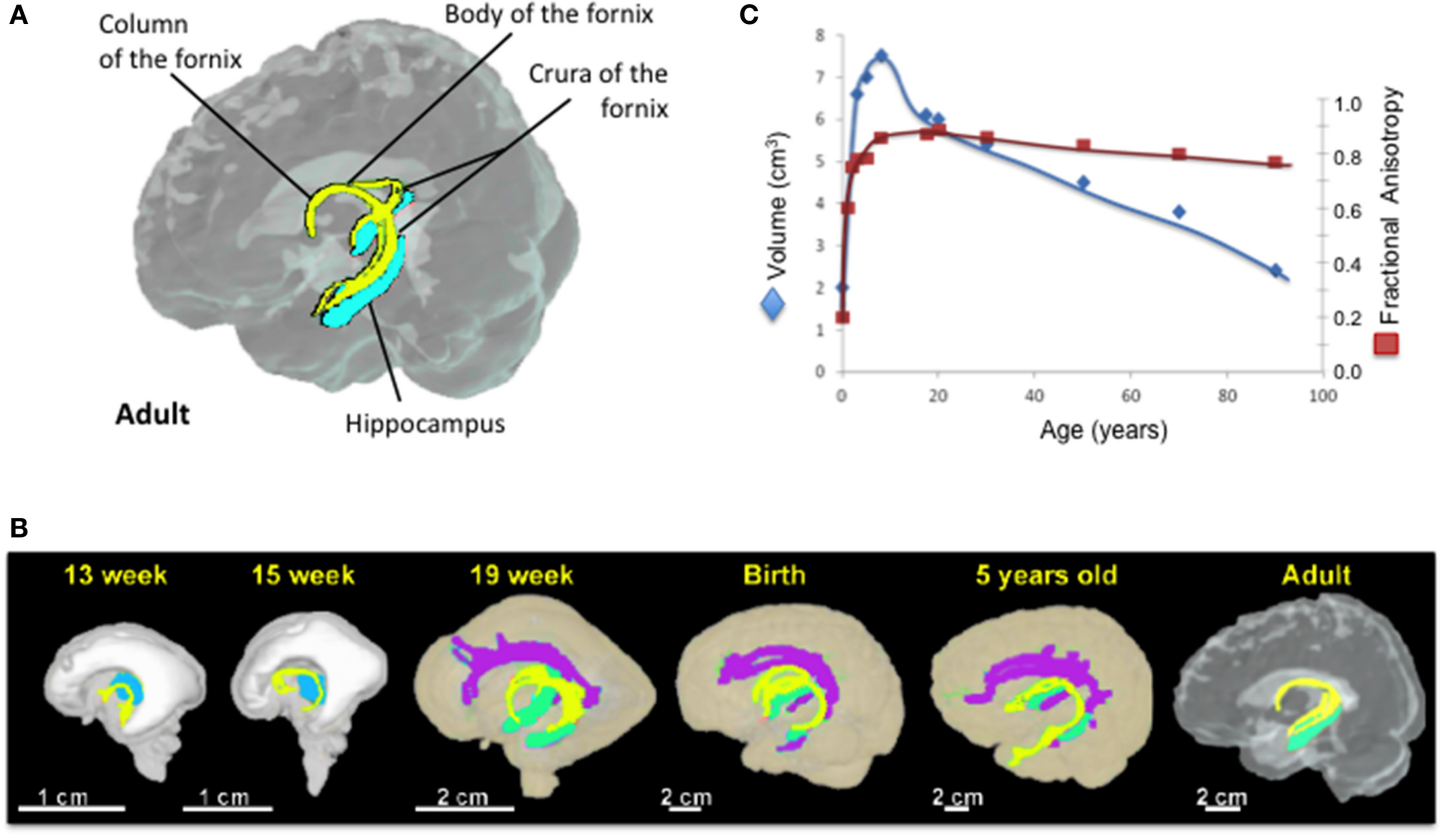
**The fornix across lifespan**. **(A)** Anatomy of the fornix in the adult brain. Courtesy of Dr. Kenichi Oishi. **(B)** 3D reconstruction of the developmental tract of the fornix from 13 weeks of gestational age to adulthood. **(C)** Developmental trajectories of forniceal volume (blue) and FA (red) from birth to 90 years old. (Adapted from Huang et al., 2006 and Huang et al., 2009). Yellow, the fornix; Green, the hippocampus; Purple, cingulum; Blue, Thalamus.

The fornix of the brain is a C-shaped structure that projects from the posterior hippocampus to the septal area and hypothalamus. As the hippocampus terminates near the splenium of the corpus callosum, the fimbria becomes a detached bundle, the crus of the fornix. The two crura merge medially to form the body of the fornix. At the interventricular foramen, the body of the fornix diverges into the two adjacent columns that pass through the middle of the hypothalamus toward the mammillary bodies (Figure 1A).

The fornix is the largest efferent pathway from the hippocampus, and belongs to the “Papez circuit,” which is also referred to as the limbic system. Forniceal fibers from the forebrain project to the anterior nucleus of the thalami, the mammillary bodies, hypothalamus, the septal nuclei and the ventral striata. Some fibers of the precommissural fornix spread beyond the septal nuclei and the ventral striata, and reach the orbital and anterior cingulate cortices. Forniceal fibers also contact the entorhinal cortex, amygdalae and back-project to the posterior cingulate gyrus (Nolte, 2009). The Papez circuit, or the limbic system, is involved in learning, memory, emotion and social behavior (King et al., 2013).

### Normal development and aging of the fornix (Table 1)

On T1-weighted MRI, the left and right columns of the fornix are difficult to delineate, and are mainly treated as a single central structure that diverges into both cerebral hemispheres. Forniceal changes are often associated with abnormalities in surrounding structures, resulting in structural distortions that are difficult to assess. However, DTI can differentiate more easily the fornix from surrounding structures, and can quantify microstructural changes within the fornix. DTI characterizes the three-dimensional diffusion of water molecules and provides information on the integrity of tissue microstructures. The fractional anisotropy (FA) value indicates the architectural degree of the tissue, which may be influenced by the amount of myelination, the coherence of axonal fibers, or a combination of both, while the mean diffusivity (MD) value is a measure of the overall averaged water diffusion within a volume of tissue. For instance, a lower than typical FA observed during development of healthy children might indicate hypomyelination or slower growth of the axons, while a decline in FA might reflect either demyelination or a decline in the number of myelinated axonal fibers, or both. An increase in MD is associated with either neuronal damage or degeneration of microstructural barriers such as cell membranes. Loss of myelin typically increases radial diffusivity (RD), whereas axial diffusivity (AxD) may be a more specific marker of axonal damage (Song et al., 2002, 2003).

**Table 1:** **Fornix metrics across the lifespan**.

**Authors**	**Subjects [age, M(male), F(female)]**	**Imaging Parameters**	**Image Analysis**	**Fornix-related Findings**
Rados et al., 2006	16 post-mortem fetal brains (10–30 weeks gestation)	T1 and T2 weighted MRI Nissl-staining	Visualization	Fornix at 10 weeks of gestational age.
Huang et al., 2006	3 post-mortem fetal brains (19–20 weeks gestation)	4.7 T (postmortem fœtus), 7 directions	Tractography, 4 tracts, 7 ROIs	Fornix such as cingulum already prominent during fetal stage, as early as 19 weeks of gestational age.
	3 female newborns	1.5 T (living subjects), 30 directions, 1.88 mm slice (newborns) and 2.3 mm slice (children)		
	3 children (5–6 years, 2M, 1F)			
Huang et al., 2009	30 post-mortem fetal brains (13–22 weeks gestation); 3 brains per week	11.7 T (13–16 weeks), 200–400 μm slice	4 tracts and 7 ROIs	Fornix is the major tract at 13 weeks of gestational age although it is a small tract in adults
		4.7 T (≥17 weeks), 300–600 μm slice, 6 directions		
Dubois et al., 2008	23 term born infants [10.3 ± 3.8 (3.9–18.4) maturational age; 12M, 11F]	1.5 T, 14–30 directions, 2.5 mm slice	Tractography (12 ROIs)	↑FA during first week of infancy
				↓MD and RD during first week of infancy
Hermoye et al., 2006	30 children [16 ± 16 months (0–4.5 years), 17M, 13F]	1.5 T, 32 directions, 1.9 mm slice (newborns) and 2.3 mm slice (children)	12 ROIs	Fornix present at birth and prominent compared to other brain structures
Douet et al., 2014	972 children [12.03 ± 3.6 (3–20) years, 509 boys, 463 girls]	3 T scanners (*n* = 10), 30 directions, 2.5 mm slice	5 ROIs	↑FA with age (max at 14.8 years) then plateau
				↑volume with age (max at 12.6 years) then decrease slightly
				↑volume α ↑episodic memory
				↑FA and ↓volume α ↓episodic memory in children with *NRG1*-TT-risk alleles for schizophrenia and psychosis
Simmonds et al., 2014	128 young adults [14.9 ± 4.2 (8–29) years, 61M, 67F]	3 T, 6 directions, 1.56 mm slice	42 ROIs	↔FA for the body portion, ↑AxD and RD (+1–2% per year) after age 20 years ↑FA with age (13.1–16.4 years, +1–2% per year) for the crescent portion
Rudebeck et al., 2009	25 Healthy Controls [25.3 ± 2.9 (22–31) years, 14M, 9F]	3 T, Diffusion-weighted imaging	TBSS, VBM, 1 ROI	↑FA α ↑episodic memory (recollection) Spatial recognition FA
Lebel et al., 2012	403 [31.3 ± 21.5 (5–83) years, 195M, 208F]	1.5 T, 6 directions, 3 mm slice	Tractography	Inverted U-shaped curve of FA with age (max at 19.5 years old); U-shaped curve for diffusivities (MD, RD, AxD) with age (min ~17.5 years old); Inverted U-shaped curve for volume (max 21.3 years)
Sala et al., 2012	84 Healthy controls [44 (13–70) years, 36M, 48F]	1.5 T, 12 directions, 4 mm slice	Automated atlas-based ROIs	Inverted U-shaped curve of FA with age; U-shaped curve for MD
				↑AxD and RD and ↓volume with age
Giorgio et al., 2010	66 adults [31M, 35F]- 35 young [23–40 years, 16M, 21F], 19 middle-age [41–60 years, 9M, 10F], 10 older [60–82 years, 6M, 4F]-	1.5 T, 60 directions, 2.5 mm	TBSS, VBM	↓volume in older adults compared to young and mid-adults
Michielse et al., 2010	69 adults [46.9 ± 17.8 (22–84) years, 17M, 52F]	1.5 T, 6 directions, 2 mm slice	9 ROIs	Linear ↓volume and FA with age
			Tractography (crus only)	Linear ↓AxD and MD with age and RD ↔
				No asymmetry with age
Lee et al., 2009	31 adults [36 (19–62) years, 15M, 16F]	3 T, 32 directions, 2.5 mm slice	14 Manual ROIs	No age-related changes in FA and ADC.
				No sex-difference
Stadlbauer et al., 2008	38 adults [49.6 ± 20.1 (18–88) years, 18M, 20F]	3 T, 6 directions, 1.9 mm slice	Tractography	↓FA with age (−2.1% per decade),
				↓number of tract
				↑MD (4.2% per decade)
Pagani et al., 2008	84 adults [44 (13–70) years, 36M, 48F]	1.5 T, 12 directions, 4 mm slice	VBM	↓volume with age
			11 clusters	
Zahr et al., 2009	24 adults- 12 young [25.5 ± 4.34 (29–33) years, 12 older adults [77.67 ± 4.94 (67–84) years-	3 T, 15 directions, 2.5 mm slice	Tractography	↓FA and ↑ADC, RD and AxD in older adults compared to young. ↑FA and ↓ADC correlate with ↑working memory, motor, problem solving scores
			8 ROIs	
Sullivan et al., 2010	120 adults [48.3 ± 14.4 (20–81) years, 55M, 65F]	1.5 T, 6 directions, 4 mm slice	Tractography	↑ADC, RD, and AxD with age
				No changes in FA
Burzynska et al., 2010	143 adults—80 young [25.7 ± 3.2 (20–32) years, 45M, 35F], 63 older [64.8 ± 2.9 (60–71) years, 34M, 29F]-	1.5 T, 12 directions, 2.5 mm slice	TBSS, VBM (body/colum and crus)	Body/column: ↓FA and ↑ diffusivities (MD, RD, and AxD) in older adults compared to young
				Crus: ↓FA and ↑RD and AxD in older adults compared to young
Jang et al., 2011	60 adults [49.2 (20–78) years, 30M, 30F]- young adults: 20–39 years, mid-adults: 40–59 years, older adults: 60–79 years-	1.5 T, 32 directions, 2.3 mm slice	Tractography	↓FA and ↑ADC with age
			3 ROIs (body, column and crus = 3parts)	↓number of tract
Sasson et al., 2013	52 adults [51 (25–82 years), 20M, 32F]	3 T, 19 directions, 2.5 mm slice	Tractography, VBA	↓FA and ↑AxD with age
Pelletier et al., 2013	129 Healthy controls [73.9 years, ≥65 years, 68M, 61F]	3 T, 21 directions, 2.5 mm slice	TBSS and 2 ROIs	↓FA with age; FA as a predictor of age
				↑FA α ↑ hippocampal volume
Vernooij et al., 2008	832 Healthy controls [73.9 ± 4.8 years, ≥55 years, 413M, 419F]	1.5 T, 25 directions, 2.5 mm slice	TBSS	↓ Volume, ↓FA, ↑AxD and RD with age
Metzler-Baddeley et al., 2011	46 adults [67.9 ± 8.6 (53–93) years, 21M, 25F]	3 T, 30 directions, 2.4 mm slice	Tractography	↓FA with age
			4 ROIs	↑FA α ↑episodic memory
Fletcher et al., 2013	102 [73 ± 6.4 years, 20 converters to MCI, and 82 non converters]	1.5 T, 6 directions, 1.5 mm slice	1 manual ROI (body only)	↓FA and volume with age
Yasmin et al., 2009	100 adults [58 ± 11 (40–84) years, 50M, 50F]	3 T, 13 directions, 2.5 mm slice	8 ROIs	↓FA and ↑MD with age

α, correlate; T, Tesla; ROI, Region of Interest; FA, fractional anisotropy; MD, Mean Diffusivity; AxD, Axial diffusivity; RD, Radial diffusivity.

VBM, voxel based morphometry; TBSS, Tract based spatial statistics.

Recently, the development of the fornix across the lifespan has become an active area of investigation because of the quality of visualization which is possible with DTI. However, only a small minority of these studies was conducted longitudinally (Table 1).

On the post-mortem human fetal brain, the fornix can be identified on MRI as early as 10 weeks of gestation (Rados et al., 2006). DTI techniques showed that the fornix is one of the most prominent tracts in the fetal brain and its entire tract is fully formed by 13 gestational weeks (Huang et al., 2006, 2009). At birth, the fornix is more prominent compared to the other brain fiber tracts and this phenotype is retained during infancy (Hermoye et al., 2006; Dubois et al., 2008). The development of fornix is thought to be completed by age 5 years (Hermoye et al., 2006; Dubois et al., 2008; Lebel et al., 2012) (Figure 1B). However, three cross-sectional (Lebel et al., 2012; Sala et al., 2012; Douet et al., 2014) and two longitudinal (Simmonds et al., 2014) DTI studies that investigated the volume and/or white matter integrity and density of the fornix showed its development through adolescence, and further age-related changes of the fornix throughout the lifespan (Figure 1C). Forniceal (body/column and crescent) FA exhibits an inverted U-shaped curve while the MD shows a U-shaped curve, and both peak at late adolescence (maximum at 19.5 years for FA and minimum at 17.8 years for MD) (Lebel et al., 2012; Sala et al., 2012). A recent longitudinal study reported no significant changes in the developmental trajectory of FA of the forniceal body/column, while FA in its crescent portion continues to increase during adolescence [13–16 years] (Simmonds et al., 2014). These findings suggest that the age-related changes of FA observed in the cross-sectional studies were primarily due to changes in the crescent rather than in the body/column of the fornix. Before peaking at late adolescence, the fornix has the steepest age-dependent increase in MD amongst all major tracts (Lebel et al., 2012; Sala et al., 2012), with AxD and RD showing more than 2% change per year (Simmonds et al., 2014). Interestingly, age-related increase of AxD, i.e., accelerated prunning, was found in the left hemisphere but not in the right hemisphere during childhood and adolescence (Simmonds et al., 2014). Similarly, asymmetric atrophy of the hippocampus and fornix were reported in several neurological disorders such as schizophrenia (Crow et al., 1989; DeLisi et al., 1997; McDonald et al., 2000; Chance et al., 2005; Mitchell and Crow, 2005; Mitelman et al., 2005), bipolar disorders (Brisch et al., 2008), temporal lobe epilepsy (Baldwin et al., 1994; Hori, 1995; Kim et al., 1995; Kuzniecky et al., 1999), and in some patients with traumatic brain injury (Tate and Bigler, 2000; Tomaiuolo et al., 2004). Therefore, finding a more sensitive neuroimaging marker to assess the forniceal lateralization, such as AxD of the fornix, may be useful for early diagnosis of these disorders.

Prior to adulthood, the forniceal volume also exhibits an inverted U-shaped curve with age, and thereafter an age-dependent decrease in the volume in both longitudinal and cross-sectional studies (Pagani et al., 2008; Giorgio et al., 2010; Michielse et al., 2010; Lebel et al., 2012; Sala et al., 2012; Fletcher et al., 2013).

During adulthood, the white matter integrity and density of the fornix typically decrease with age across DTI studies (Stadlbauer et al., 2008; Lee et al., 2009; Yasmin et al., 2009; Michielse et al., 2010; Sullivan et al., 2010; Lebel et al., 2012; Sala et al., 2012; Fletcher et al., 2013; Sasson et al., 2013). The majority of the studies found age-related decreases of the forniceal FA (Stadlbauer et al., 2008; Yasmin et al., 2009; Zahr et al., 2009; Burzynska et al., 2010; Michielse et al., 2010; Jang et al., 2011; Metzler-Baddeley et al., 2011; Lebel et al., 2012; Sala et al., 2012; Fletcher et al., 2013; Pelletier et al., 2013; Sasson et al., 2013), and only two studies showed no changes with age (Lee et al., 2009; Sullivan et al., 2010). Findings on the diffusivities (MD, AxD and RD) are less consistent and varied depending on the region of interest (crus, body/column or the entire fornix). While the majority of the studies found diffusivities (MD, AxD and RD) of the fornix increase with age (Stadlbauer et al., 2008; Yasmin et al., 2009; Zahr et al., 2009; Burzynska et al., 2010; Sullivan et al., 2010; Jang et al., 2011; Lebel et al., 2012; Sala et al., 2012; Sasson et al., 2013; Simmonds et al., 2014), several studies reported either age-related decrease of MD and AxD (Michielse et al., 2010) or no changes in the fornix with age for MD (Lee et al., 2009) and RD (Michielse et al., 2010) across the age span of 10 to 80 years. White matter maturation follows sex-specific differential trajectories (Westerhausen et al., 2004; Schmithorst et al., 2008; Asato et al., 2010). Girls showed maturation of white matter integrity earlier than boys (Asato et al., 2010). In particular, girls showed greater age-dependent increase of MD in associative regions compared to boys. Furthermore, tendencies for age-related increase of FA were found in the right hemisphere for girls but in the left hemisphere for boys. These sex-specific brain differences parallel the pubertal changes that occur during adolescence, suggesting that hormonal changes might influence white matter maturity. However, the few studies that examined the relationships between physical pubertal maturity and circulating hormones on white matter maturation were underpowered by sample size(Peper et al., 2008, 2009). Nevertheless, discrepancies on diffusivities between DTI studies are not due to differences in age range and sex distribution, since they are similar across all of these studies. The image processing methods for these studies are also similar between those that showed conflicting results. Therefore, sample size or inter-subject variations might have contributed to the different findings regarding the age-related changes in FA and MD.

Overall, the fornix is one of the earliest white matter tracts to mature. After its maturation peaks during late adolescence, the fornix begins to “atrophy” throughout the remainder of the lifespan. However, “pruning” rather than degenerative processes likely contribute to the early decreases in forniceal volume. More detail anatomical assessments of the fornix (column, body, and crus) and more systematic evaluations across a larger age range, followed longitudinally, are needed to better characterize the developmental trajectories of the fornix.

#### Relationship with cognition

Fibers from the fornix comprise the main cholinergic input to the hippocampi and major efferent pathways from the hippocampi to the anterior thalamic nuclei, mammillary bodies, striata, and prefrontal cortices. These anatomical connections are involved in memory networks, which demonstrate that the fornix plays a critical and central role in memory tasks, particularly episodic memory. However, few studies investigated the relationships between forniceal metrics and memory tasks during typical development and aging. During childhood [3–20 years], larger forniceal volume was correlated with better episodic memory scores in healthy children. But this relationship was reversed in those carrying the *NRG1*-T-risk alleles for schizophrenia and psychosis (Douet et al., 2014). During young adulthood [22–31 years], greater FA in the fornix was associated with better episodic memory scores, especially with spatial recognition (Rudebeck et al., 2009). Similarly, across studies of young adults and older adults, forniceal FA correlated positively with working memory (Zahr et al., 2009), episodic memory (Rudebeck et al., 2009), and with both verbal and visual recall tasks (Rudebeck et al., 2009; Zahr et al., 2009; Metzler-Baddeley et al., 2011). A 4-year longitudinal follow-up study of healthy older adults found that lower forniceal volume and higher AxD at baseline predicted conversion to cognitive impairments (mild cognitive impairment or dementia) (Fletcher et al., 2013). Therefore, volumetric and white matter changes of the fornix appear to be effective biomarkers to validate or corroborate with memory performance across the lifespan, and to predict hippocampal function (Aggleton et al., 2000; Rudebeck et al., 2009; Fletcher et al., 2013; Pelletier et al., 2013).

### Fornix as a predictor of memory deficits

Early studies in humans did not report associated memory deficits after lesion of the fornix (Garcia-Bengochea and Friedman, 1987). More recent studies, however, consistently reported deficits in several cognitive abilities, especially in episodic memory, in patients with injuries to the fornix (Gaffan et al., 1991; Squire and Zola-Morgan, 1991; Aggleton et al., 2000). Moreover, as part of the limbic system, fornix degeneration may precede hippocampal dysfunction, and may predict conversion to cognitive impairment better than hippocampal atrophy (Fletcher et al., 2013). Hence, assessments of the fornix have recently become a major research focus in determining its role in neurological disorders that are associated with memory impairments.

#### Alzheimer disease and dementia syndromes (Table 2)

Alzheimer disease (AD) and mild cognitive impairment (MCI) can be distinguished from normal aging by the different clinical syndromes (Petersen et al., 2001). MCI includes amnestic MCI (aMCI) and non-amnestic MCI (naMCI) (Petersen, 2004), depending on the memory impairment features. While naMCI patients tend to develop frontotemporal dementia or other types of dementias with broader cognitive deficits, aMCI patients are at risk for Alzheimer's disease (Mielke et al., 2014). In the US population, the prevalence of MCI ranges from 3 to 19% depending on the studies. About 40% of MCI patients will develop AD or other dementias, while most of MCI patients stay stable, and some even revert to a healthy control diagnosis (Mielke et al., 2014). Therefore, understanding prodromal AD and predicting accurately when MCI will convert to dementia can lead to early diagnosis and prevention of dementia when effective preventive strategies become available.

**Table 2:** **Forniceal macro- and micro-structure alterations in patients with Alzheimer's disease and mild cognitive impairments**.

**Authors**	**Subjects [Mean age ± *SD* (age range), Male, Female]**	**Image Acquisition**	**Image Analysis**	**Fornix-related Findings**
**STRUCTURAL MRI STUDIES**				
Callen et al., 2001	40 AD [69.1 ± 7.3 (54.5–80) years, 20M, 20F]	1.5 T, T1-weighted MRI, 1.5 mm slice	ROI	Volume: AD < HC
	40 HC [70.4 ± 6.3 (55.8–80.6) years, 20M, 20F]			
Copenhaver et al., 2006	16 AD [75.6 ± 6.9 (63–86) years, 7M, 9F]	1.5 T, T1-weighted MRI, 1.5 mm slice	ROI (crus)	Volume: AD < HC
	20 CC [73.9 ± 6.6 (63–86) years, 6M, 14F]			↓volume with age in all groups
	20 MCI [69.6 ± 6.2 (63–86) years, 10M, 10F]			
	20 HC [71.3 ± 5.7 (63–86) years, 6M, 14F]			
**DIFFUSION TENSOR STUDIES**				
Ringman et al., 2007	12 FADmc [35 ± 6.4 years, 2M, 10F]	1.5 T, 6 directions	ROI	Area: FADmc < FADnc
	8 FADnc [36 ± 6.2 years, 1M, 7F]			FA: FADmc < FADnc
				↓ FA ∝ ↓all NPTs and ↑ AD severity
Stricker et al., 2009	16 AD [77.3 ± 9.0 years, 8M, 8F]	3 T, 15 directions, 3 mm slice	TBSS	FA: AD < HC
	14 HC [77.4 ± 8.1 years, 5M, 9F]			
Mielke et al., 2009	25 AD [75.6 ± 7.0 years, 18M, 7F]	3 T, 30 directions	ROI (body)	No difference longitudinally (3 months)
	25 MCI [75.8 ± 5.3 years, 18M, 7F]	2.2 mm slice	3-month follow-up	Cross-sectionally
	25 HC [74.3 ± 7.1 years, 11M, 14F]			FA: MCI > AD < HC
				In MCI and AD: ↓FA ∝ ↓ memory scores (on CVLT) and ↓CDR
Sexton et al., 2010	7AD [68.1 ± 9.6 years, 5M, 2F]	1.5 T, 51 directions, 2.8 mm slice	TBSS and ROIs	↑FA (Left_crus), ↓AxD (Left_crus), ↓MD (crus) and RD (crus) α ↑episodic memory factor (CVLT-R, HVLT-R, RCFT)
	8 MCI [73.0 ± 7.5 years, 3M, 5F]		(Body and crus)	
	8HC [77.1 ± 4.6 years, 3M, 5F]			
Zhuang et al., 2010	96 aMCI [79.57 ± 4.71 (70–90) years, 57M, 39F]	3 T, 6 directions	TBSS	FA: aMCI < HC
	69 naMCI [77.62 ± 4.49 (70–90) years, 21M, 48F]	3.5 mm slice		FA_:_discriminated ~70% (aMCI vs. HC)
	252 HC [77.87 ± 4.52 (70–90) years, 106M, 146F]			
Kantarci et al., 2011	149 MCI/71 HC [median 79 (52–95) years]	3 T, 21 directions	ROIs and VBM	↑FA ∝ ↑language function, ↑visual-spatial processing
		3.3 mm slice		
Liu et al., 2011b	17 AD [76 ± 7 years, 6M, 11F]	1.5 T, 30 directions	TBSS	FA: AD < HC
	27 MCI [75 ± 6 years, 15M, 12F]	5 mm slice		FA: AD < MCI in the right fornix
	19 HC [75 ± 6 years, 11M, 8F]			
Cui et al., 2012	79 aMCI [79.42 ± 4.71 years, 49M, 30F]	3 T, 6 directions	ROI	Crus discriminates between MCI and HC
	204 HC [77.65 ± 4.37(67–90) years, 85M, 119F]	3.5 mm		
Hattori et al., 2012	20 AD [74.6 ± 5.7 years, 10M, 10F]	1.5 T, 13 directions	Tractography	Volume: iNPH < AD < lHC
	22 iNPH [77.3 ± 4.9 years, 10M, 12F	3 mm slice		FA: iNPH < HC; AD < HC
	20 HC [73.9 ± 6.0years, 7M, 13F]			fornix differentiated iNPH from AD
Huang et al., 2012	26AD [70.8 ± 8.2 years, 15M. 11F]	3 T, 30 directions	ROI	FA: AD < HC
	11aMCI [69.1 ± 7.3 years, 5M, 6F]	2. mm slice		MD and RD: AD > HC; No group difference in AxD
	24HC [69.5 ± 7.1 years, 10M, 14F]			
Metzler-Baddeley et al., 2012	25 MCI [76.8 ± 7.3 years, 14M, 11F]	3 T, 30 directions	Tractography	No correlation between FA and episodic memory; ↓FA with age
	20 HC [74 ± 6.5years, 10M, 10F]	2.4 mm slice	ROI	
Mielke et al., 2012	23 aMCI [75.6 ± 5.5 years, 16M, 7F]	3 T, 32 directions	ROI(body) 3-, 6-, 12-month and 2.5 yrs follow-ups	↓ FA correlated with↓ memory (CVLT) and ↓CDR
		2.2 mm slice		↑ MD, AxD, RD correlate with ↓ memory
				FA and MD predicted AD progression
				Longitudinally: no difference in FA or diffusivities
Oishi et al., 2012	25 AD [75.6 ± 6.9 years, 18M, 7F]	3 T, 30 directions	ROI	Cross-sectionally: FA: AD < MCI or HC
	25 aMCI [75.8 ± 5.2 years 18M, 7F]	2.2 mm slice	6- and 12 month follow-ups	↓ FA ∝↓memory performance (WMS delayed recall, CVLT)
	25 HC [74.3 ± 7.1 years, 11M, 14F]			FA preded conversion from HC to aMCI, and from aMCI to AD
				Longitudinally: no difference in FA or diffusivities
Douaud et al., 2013	22 sMCI [69 ± 9 years, 11M, 11F]	3 T, 30 directions	TBSS	Volume: pMCI < sMCI
	13 pMCI [76 ± 6 years, 3M, 10F]	3 mm slice		FA: pMCI < sMCI; MD: pMCI > sMCI
				↓FA ∝ ↑ MD ∝ ↓vol
Nowrangi et al., 2013	25 AD [75.6 ± 6.9 years, 18M, 7F]	3 T, 32 directions	ROI	FA: AD < HC/MCI
	25 aMCI [75.8 ± 5.2 years 18M, 7F]	2.2 mm slice	6- and 12 month follow-ups	MD: AD > HC/MCI
	25 HC [74.3 ± 7.1 years, 11M, 14F]			↑ MD in all subjects over 12 month (greater ↑ MD over 6 month in MCI compared to HC)
Fletcher et al., 2013	102 [73 ± 6.4 years, 20 converters to MCI, and 82 non–converters]	1.5 T, 6 directions, 1.5 mm slice	1 manual ROI (body only)	↓FA and volume with age
Canu et al., 2013	22 EOAD [59.4 ± 4.6 (48–68)years, 11M, 11F]	3 T, 35 directions	ROI	FA: EOAD < Younger HC
	24 Younger HC [59.1 ± 2.7 (51–64) years, 12M, 12F]	2.3 mm slice	VBM	MD and RD: EOAD > Younger HC
	35 LOAD [75.4 ± 4.6 (68–84)years, 12M, 23F]			
	16 Older HC 73.1 ± 4.3 (67–81) years, 6M, 10F]			
Zhuang et al., 2013	27 “late” aMCI [81.0 ± 4.6 (74.0–88.8) years, 18M, 9F]	3 T, 32 directions	TBSS	FA: late aMCI < HC(in left fornix)
	39 “early” aMCI [74 ± 5.3 (72.9–90.7) years, 24M, 15F]	2.5 mm slice	ROI	AxD, RD and MD:late or late aMCI > HC (entire fornix)
	155 HC [79.1 ± 4.4 (72.5–90.5) years, 61M, 94F]			↓FA and ↑MD ∝ ↓ episodic memory

MRI, Magnetic Resonance Imaging; T, Tesla; TBSS, Tract-based spatial statistic; VBA, Voxel-based analysis; ROI, Region of Interest; FA, Fractional Anisotropy; MD, Mean Diffusivity, AxD, Axial Diffusivity; RD, Radial Diffusivity.

EOAD, early-onset Alzheimer's disease; LOAD, late-onset Alzheimer's disease; HC, Healthy controls; naMCI, non-amnesic; MCI;AD, Alzheimer disease; iNPH, idiopathic normal pressure hydrocephalus; FAD, familial Alzheimer's disease; FADmc, familial Alzheimer's disease mutation carriers, FADnc, familial Alzheimer's disease non-carriers; pMCI, amnestic MCI patients who progressed to probable AD no earlier than 2 years after their baseline scan; sMCI, amnestic MCI patients who were clinically stable i.e., did not develop AD for at least 3 years following their first evaluation.

WMS–R, Wechsler Memory Scale–Revised; CDR, Clinical Dementia Rating; RCFT, Rey Complex Fig Test; HVLT-R, Hopkins Verbal Learning Test-Revised.

The neuropathology of AD is characterized by the presence of extracellular beta-amyloid plaques and intracellular neurofibrillary tangles that both lead to neuronal dysfunction and apoptosis (Bossy-Wetzel et al., 2004). Neurofibrillary tangles result from the intracellular oligomerization of the microtubule-associated protein Tau. The deposition of neurofibrillary tangles begins primarily in the limbic system structures, initially in the entorhinal cortex and the medial temporal regions, then progressively spread across the cerebral cortex. Hippocampal and entorhinal cortical atrophy assessed with MRI is well documented in patients with AD (Teipel et al., 2013), and in many with MCI (Pihlajamaki et al., 2009). Furthermore, this observation has extended the investigation of all limbic structures in relation to disease progression and cognitive performance.

The fornix is atrophied in MCI and AD patients compared to healthy controls, (Callen et al., 2001; Copenhaver et al., 2006; Ringman et al., 2007; Hattori et al., 2012) as confirmed by a longitudinal follow-up study (Douaud et al., 2013). Furthermore, in a large cohort of 79 aMCI and 204 healthy controls (HC), the volume of the crus of the fornix more specifically discriminated between MCI and HC (Cui et al., 2012).

Decreased FA of the fornix, on DTI, was found to be more sensitive than decreases in volume and/or area, on structural MRI, for predicting AD progression, since decreased FA preceded the atrophy more than two years prior to conversion from MCI to AD (Douaud et al., 2013). AD patients had lower FA (Liu et al., 2011b; Metzler-Baddeley et al., 2012) and higher MD and RD in the fornix compared to healthy controls (Mielke et al., 2009; Stricker et al., 2009; Liu et al., 2011b; Hattori et al., 2012; Huang et al., 2012; Oishi et al., 2012; Nowrangi et al., 2013; Zhuang et al., 2013), and at disease onset as defined by comparison between MCI and/or early onset AD patients (Mielke et al., 2009; Zhuang et al., 2010; Liu et al., 2011b; Oishi et al., 2012; Canu et al., 2013; Douaud et al., 2013; Nowrangi et al., 2013). A similar phenotype of lower FA in the fornix was found also in patients with genetically inherited dementias in comparison to controls (Ringman et al., 2007). Longitudinal studies showed that the magnitude of age-related changes of DTI metrics is similar between AD, MCI and healthy controls (Mielke et al., 2009, 2012; Oishi et al., 2012), suggesting that abnormal forniceal FA and MD are likely to predict convertion from MCI to AD.

Lower FA and higher diffusivity metrics in the fornix were associated also with worse performance on short- and long-term memory tasks and with clinical dementia evaluations in AD and MCI patients (Ringman et al., 2007; Mielke et al., 2009, 2012; Kantarci et al., 2011; Zhuang et al., 2013), as well as in healthy controls (Sexton et al., 2011; Oishi et al., 2012; Nowrangi et al., 2013). These cognitive measures showed deficits in verbal memory (i.e., California Verbal Learning Test, Hopkins Verbal Learning Test) and visual memory (Rey-Osterrieth Complex Figure Test), as well as in more global measures (MMSE and Clinical Dementia Rating).

Therefore, measurements of macro- and micro-structural changes in the fornix may provide preclinical surrogate markers to predict the development of Alzheimer disease and allow early treatment in these patients.

#### Schizophrenia (SCZ) (Table 3)

Clinical signs, brain imaging and genetic studies all contributed to the hypothesis that schizophrenia and psychiatric diseases are neurodevelopmental disorders (Rapoport et al., 2012) with neurodegenerative components (Vita et al., 2012). In addition, findings from postmortem and neuroimaging studies suggest that white matter maturation and myelination processes are disrupted in schizophrenia, which might trigger its symptoms (Heckers et al., 1991; Arnold et al., 1995) or lead to age-related white matter loss and cognitive decline (Chang et al., 2007; Kochunov and Hong, 2014). Brain abnormalities in SCZ patients occur in the paralimbic and temporolimbic regions (Kasai et al., 2003), which are involved in episodic memory. Incidentally, episodic memory impairment is one of the most consistent phenotype for schizophrenia (Schaefer et al., 2013). Since the fornix is part of the limbic system, and is involved in episodic memory, it has been evaluated with histopathology and brain imaging in SCZ patients.

**Table 3:** **Forniceal macro- and micro-structure alterations in schizophrenia and psychiatric disorders**.

**Authors**	**Subjects [Mean age ± *SD*, range, (Male/Female)]**	**Image Acquisition**	**Image Analysis**	**Fornix-related Findings**
Chance et al., 1999	29 SCZ [70 ± 13.8 years, 16M, 13F]	Post mortem brain	Palmgren's silver stain for nerve fibers	Fiber density: men < women
	33 HC [69.45 ± 12.7 years, 19M, 14F]	Parrafin wax		Fiber density in men: SCZ > HC in the left fornix only
		5 μm section		No group difference in the numbers of fibers
Brisch et al., 2008	19 SCZ [51.37 ± 7.85 years, 11M, 8F]	Post mortem brain	Nissl and myelin-stained	No differences in volume and mean cross-sectional areas
	9 bDep [51.78 ± 11.90 years, 6M, 3F]	20 μm section		
	7 uDep [46.71 ± 14.31 years, 2M, 5F]			
	14 HC [53.64 ± 9.61 years, 8M, 4F]			
Davies et al., 2001	17 SCZ [16.9 ± 0.4 (14.83–20.5) years, 11M, 6F]	1.5 T	ROI (body)	Area: SCZ > HC (+39.69%)
	9 PsyC [16.25 ± 0.5 (12.7–17.8) years, 6M, 38F]	MRI		Area: SCZ > PsyC (+26.23%)
	8 HC [16.9 ± 0.58 (14–18.3) years, 4M, 4F]	1.5 mm slice		Area: HC = PsyC
Zahajszky et al., 2001	15 SCZ [37.6 ± 9.3 (20–54) years]	1.5 T	ROI (body and crus)	No difference in volume between groups.
	15 matched HC [37.9 ± 8.8 (23–54) years]	MRI		No association between volume and illness or between volume and cognitive/clinical measures.
	Only men	6 directions		
		3 mm slice		
Abdul-Rahman et al., 2011	33 SCZ [39.4 ± 8.82 years, 24M, 7F]	3 T	ROI	FA: SCZ < HC
	31 HC [35.4 ± 8.82 years, 25M, 8F]	15 directions	Tractography	RD: SCZ > HC, no difference in AxD
		3 mm slice		Specific loci of FA reduction within the fornix
				in SCZ, ↓FA α ↑psychopathology
Davenport et al., 2010	15 SCZ_onset [10–20 years, 8M, 7F]	3 T	VBA	In left posterior fornix:
	14 ADHD [10–20 years, 12M, 2F]	12 directions		FA: SCZ_onset < HC and ADHD < HC
	26 HC [10–20 years, 16M, 12F]	2 mm slice		
Fitzsimmons et al., 2009	36 SCZ [39.89 ± 9.06 years]	1.5 T	Tractography	FA: SCZ < HC
	36 HC [39.59 ± 9.32 years]	6 directions	ROI	In HC: ↑FA α ↑ visual and verbal memory tasks, recall and recognition.
	Only men	4 mm slice		In SCZ: no correlations
Fitzsimmons et al., 2014	21 FES [21.71 ± 4.86 years, 16M, 5F]	3 T	Tractography	FA: FES < HC
	22 HC [21.23 ± 3.29 years, 13M, 9F)	51 directions	ROI	MD, RD and AxD: FES > HC
		Slice not reported		MD (left) < MD (right) in FES only
				No correlation between DTI metrics and clinical characteristics
Kendi et al., 2008	15 SCZ [14.5 ± 2.6 (8–19 years), 7M, 8F)]	3 T	ROI	Volume: SCZ < HC (-11%)
	15HC [15.1 ± 2.5 (8–19 years), (8M, 7F]	12 directions		No changes in FA
		2 mm		
Kuroki et al., 2006	24SCZ [40.3 ± 8.5 years (24–52 years)]	1.5 T	ROI	FA: SCZ < HC (-7.5%)
	31HC [40.6 ± 8.7 years (23–54 years)]	6 directions		MD: SCZ > HC (+6.7%)
	Only men	4 mm slice		Volume: SCZ < HC (-15.5%)
				↓FA α ↑medication dosage
				↓cross-sectional area α ↓global attention scores
				↓cross-sectional area α ↓hippocampal volume
Lee et al., 2013	17 FES [21.5 ± 4.8 (18–30 years), 13M, 4F]	3 T	TBSS	FA: FES < HC
	17 HC [23.1 ± 3.5 (18–30 years), 12M, 5F]	51 directions	ROI	In the right fornix only, ↓FA α ↓reading scores
		1.7 mm		No effect of medication on FA in FES group
Luck et al., 2010	32 FES [23.6 ± 0.7 years, 22M, 10F)]	1.5 T	Tractography	FA: FES < HC
	25 HC [24.5 ± 0.8 years, (13M, 12F]	60 directions		
		4.4 mm slice		
Nestor et al., 2007	21 SCZ [39.79 ± 9.16 (18–55 years)]	1.5 T	ROI	In SCZ: ↓FA α ↓scores for memory(↓DPT)
	24 HC [40.64 ± 9.38 (18–55 years)]	6 directions		In HC: ↑FA α ↑scores for memory (↑DPT, verbal memory and recall)
	Only men	4 mm slice		
Takei et al., 2008	31SCZ [33.8 ± 9.0 (22–55 years), 12M, 19F)]	1.5 T	Tractography	FA: SCZ < HC
	65 HC [34.7 ± 9.7 (21–54 years), 24M, 41F]	6 directions	ROI	MD: SCZ > HC
		Slice not reported		No lateralization.
				In SCZ only: ↑MD_left α ↓verbal learning scores and ↑MD_right α ↓category fluency test performance
Smith et al., 2006	33 SCZ, 15 MS, Not reported	1.5 T	TBSS	FA: SCZ < HC
		10 directions		
		2.5 mm		
Maier-Hein et al., 2014	20 BPD [16.7 ± 1.6 (14–18 years)]	3 T	TBSS	FA: BPD < HC = CC
	20 mixed psychosis diagnoses (CC) [16.0 ± 1.3 (14–18 years)]	12 directions	ROI	
	20 HC [16.8 ± 1.2 (14–18 years)]	2.5 mm slice		
	Only women			

α: correlate.

MRI, Magnetic Resonance Imaging; T, Tesla; TBSS, Tract-based spatial statistic; VBA, Voxel-based analysis; ROI, Region of Interest; FA, Fractional Anisotropy; MD, Mean Diffusivity, AxD, Axial Diffusivity; RD, Radial Diffusivity.

HC, Healthy controls; SCZ, schizophrenic patients; PsyC, psychiatric controls non–schizophrenics: ADHD, Attention deficit hyperactivity disorder; uDep, unipolar Depression, bDep, bipolar depression; BPD, bipolar disorder; FES, first episode schizophrenia.

DPT, Doors and People Test.

Histopathologic studies showed that SCZ men, but not women, had greater than normal fiber density in the left fornix, suggesting sex and hemisphere specific alterations in the myelination of the fornix in schizophrenia (Chance et al., 1999). However, the fornix volume and cross-sectional area were found to be similar between SCZ patients and healthy adult controls in postmortem brain tissues (Brisch et al., 2008), and in an *in vivo* MRI study (Zahajszky et al., 2001). In contrast, larger fornices were found on MRI of adolescent SCZ (ages 16–17 years, both males and females) compared to healthy controls and to patients with other serious psychiatric disorders (Davies et al., 2001). These variable findings regarding the forniceal volume might have resulted from the different subject populations and the less well defined fornix structures on these earlier structural MRI studies.

Findings on the fornix measurement have been more consistent across DTI studies. Using tractography, forniceal bundle volume in SCZ adolescents and adults were smaller [−11–16%] than in healthy controls (Kuroki et al., 2006; Kendi et al., 2008). The various DTI studies and approaches, using tractography, regions of interest (ROI) and tract-based spatial statistics (TBSS), consistently showed that FA of the fornix is lower in SCZ patient compared to healthy control. The lower than normal FA appears early at the onset of SCZ, which typically occurs just before adolescence (Davenport et al., 2010). This phenotype was reported in adolescent patients with their first episode of SCZ (Lee et al., 2009), in SCZ young adults (Luck et al., 2010; Fitzsimmons et al., 2014) and in mid-life SCZadults (Kuroki et al., 2006; Takei et al., 2008; Fitzsimmons et al., 2009; Abdul-Rahman et al., 2011), suggesting that lower than “normal” FA is a stable marker for SCZ that is retained throughout the lifespan. Lower FA in SCZ patients is frequently accompanied by either higher MD (Kuroki et al., 2006; Takei et al., 2008), RD (Abdul-Rahman et al., 2011) or both (Fitzsimmons et al., 2014). Findings on AxD are less consistent. Two studies showed no changes in AxD between SCZ patients and healthy controls (Kendi et al., 2008; Abdul-Rahman et al., 2011), whereas another study found higher AxD along with higher MD and RD in young adults with first episode schizophrenia (Fitzsimmons et al., 2014). The higher RD was suggested to be a marker of myelin disruption, higher MD a marker of atrophy, while AxD may reflect axonal disruption (Song et al., 2005). Therefore, the lower FA and higher diffusivities involving all three measures (MD, RD and AxD) possibly reflect alterations in both myelin and axons. These alterations are notable in the fornix already at illness onset, but the causative mechanism is not yet defined. Moreover, the functionality of the forniceal changes and their impacts on the limbic network is still unclear. Some studies found no association between forniceal metrics and either cognitive or clinical measures (Zahajszky et al., 2001; Fitzsimmons et al., 2009, 2014; Lee et al., 2013), while others reported that lower FA correlated with greater psychopathology (Abdul-Rahman et al., 2011) and higher medication dosage in SCZ patients (Kuroki et al., 2006). Lower FA and/or higher MD was further associated with greater episodic memory impairments (verbal and visual memory tests) in SCZ patients (Nestor et al., 2007; Takei et al., 2008; Lee et al., 2013). In healthy controls, these correlations between FA and visual and verbal memory tasks were also observed (Nestor et al., 2007; Fitzsimmons et al., 2009).

In conclusion, abnormalities in the fornix are found in SCZ patients and are most likely due to degeneration, involving both axonal injury and demyelination, of the fornix. To some extent, these microstructural abnormalities in the fornix may serve as an imaging marker for disease severity in schizophrenia, although it remains unclear whether these changes in the fornix contribute to disruption of the limbic networks and to hippocampal atrophy. In addition, DTI metrics (FA and MD) appear to be sensitive indicators of injury to the fornix and subsequent memory deficits in SCZ patients. Further investigations using these metrics, in addition to other imaging modalities (e.g., evaluating brain network connectivities), are needed to follow patients longitudinally from the prodromal period to the first episodes to understand further the evolution of the neuropathology of schizophrenia.

#### Multiple Sclerosis and other neurodegenerative diseases (Table 4)

Multiple Sclerosis (MS) is an autoimmune demyelinating disease that is characterized by the infiltration of macrophages and T-cells that activate glia and microglia, which lead to fulminant neuroinflammation and intense demyelination of nerve fibers (Pivneva, 2008). About half of the MS patients develop cognitive deficits and most frequently, episodic memory deficits (Brissart et al., 2011). As parts of the limbic system, both the hippocampus and the fornix were often found affected in MS patients. Compared to healthy controls, MS patients had lower magnetization transfer ratio (MTR) in the right fornix, but this abnormality in the fornix did not correlate with cognitive performance (Ranjeva et al., 2005). Using TBSS, tractography or ROI, MS patients consistently showed lower FA with higher MD and RD in the fornix than healthy controls across studies and during adulthood (Smith et al., 2006; Dineen et al., 2009, 2012; Roosendaal et al., 2009; Fink et al., 2010; Kern et al., 2012; Koenig et al., 2013; Syc et al., 2013). Findings on AxD in the fornix were less consistent and less systematically investigated. Forniceal AxD showed either no group differences (Dineen et al., 2012) or higher values in MS compared to healthy controls (Roosendaal et al., 2009; Syc et al., 2013). In most of these studies, MS patients with lower FA and higher diffusivity metrics in the fornix had poorer performance in verbal and visual memory or recall and greater episodic memory impairments (Brief Visual Memory Test-Revised) (Dineen et al., 2009, 2012; Koenig et al., 2013, 2014; Syc et al., 2013). Moreover, these forniceal DTI metrics correlated with Expanded Disability Status Scale (EDSS) and disease duration in these MS patients (Syc et al., 2013; Koenig et al., 2014).

**Table 4:** **Forniceal macro- and micro-structure alterations in multiple sclerosis and other neurodegenerative diseases**.

**Authors**	**Subjects [Mean age ± *SD* (range), M (male), F (female)]**	**Image Acquisition**	**Image Analysis**	**Fornix-related Findings**
**MULTIPLE SCLEROSIS (MS)**				
Ranjeva et al., 2005	18 CISSMS [29.3 ± 7 years, 2M, 16F]	1.5 T	MTR	MTR (right fornix): MS < HC
	18 Healthy controls [25.27 ± 6.3 years, 2M, 16F]	5 mm slice		
Dineen et al., 2009	37 MS [43.5 (31.1–56.3) years, 11M, 26F]	3 T	TBSS	FA: MS < HC
	25 HC [36.4 (28.2–55.3) years, 9M, 16F]	15 directions		↓FA in the left fornix α ↓episodic memory scores (CVLT and BVRT)
		2.5 mm slice		
Dineen et al., 2012	34 relapsing-remitting MS [42.6 (31.1–56.1) years, 11M, 13F]	3 T	ROI	FA: MS < HC
	24 HC [38.7 (28.3–55.3) years, 9M, 15F]	15 directions		RD: MS > HC; no group difference in AxD
		2.5 mm slice		↓FA α ↓episodic memory scores (CVLT and BVRT)
Fink et al., 2010	50 MS [43.3 ± 9.3 (20–65) years, 10M, 40F]	1.5 T	Tracto-graphy	FA: MS < HC
	20 HC (41.3 ± 10.1 (20–56) years]	30 directions	ROI	RD: MS > HC in left fornix only
		1 mm slice		In MS, ↑ RD (Right fornix) α ↓episodic long-term memory (CVLT_recognition)
Kern et al., 2012	18 MS [42.1 (23–54.5 years), 14M, 4F]	3 T	TBSS	FA: MS < HC
	16 HC [35.2 (24–50.3 years), 14M, 2F]	12 directions		In MS: ↓FA α ↓verbal memory performance
		3 mm slice		
Koenig et al., 2013	40 MS [42.55 ± 9.1 (32–52 years), 11M, 29F]	3 T	ROI	FA: MS < HC
	20 HC [41.35 ± 9.7 (32–52 years), 7M, 13]	71 directions		RD and MD: MS > HC
		1 mm slice		In MS: ↑RD, MD and ↓FA(Left-fornix) α ↓episodic memory (BVMT-R scores)
				No group difference in volume
Koenig et al., 2014	52 MS [44.27 ± 8.9 (32–52 years), 16M, 36F]	3 T	ROI	Volume: MS < HC
	20 HC [41.35 ± 9.7 (32–52 years), 7M, 13F]	71 directions		In MS: ↓FA and volume (Left-fornix)and ↑MD, AxD and RD α ↓episodic memory (BVMT-R and SDMT)
		1 mm slice		↑MD, RD, and AxD (Right-fornix) and ↓volume α ↑EDSS
				In MS: ↑FA and ↓MD, RD, AxD α ↑hippocampal volume
				No correlation in HC
Roosendaal et al., 2009	30 MS [40.6 ± 9.1, 11M, 19F]	1.5 T	TBSS	FA: MS < HC
	31 HC [40.6 ± 9.9 years, 10M, 21F)]	61 directions	ROI	RD and AxD: MS >HC
		3 mm slice		No correlation between FA and EDSS
Syc et al., 2013	64 RRMS [39 ± 11 (32–52 years), 23M, 41F]	3 T	Tractography	FA: MS < HC (-19%)
	24 SPMS [55 ± 8 (32–52 years), 7M, 17F]	MTR	ROI	MD, RD and AxD: MS > HC (+13%)
	13 PPMS [56 ± 7 (32–52 years), 7M, 6F]	1.5 mm slice		↓FA and ↑MD, RD, AxD α ↑EDSS and ↑disease duration
	16 HC [40 ± 9 (32–52 years), 5M, 11F]			↓FA and ↑MD, RD α ↓PASAT-3 scores
				↓FA and ↑MD, RD and AxD α ↑9-HPT times
**PARKINSON'S DISEASE (PD)**				
Matsui et al., 2006	11 PD with EDS (ESS > 10) [72.2 ± 7.2 years, 8M, 3M]	1.5 T	5 manual ROIs	FA: PD with EDS < PD without EDS or controls
	26 PD without EDS [71.2 2419.2 years, 23F, 3M]	6 directions		FA α with Epworth Sleepiness Scale (ESS)
	10 controls [72.4 ± 6.4 years, 7M/3F]	4 mm slice		
Matsui et al., 2007	14 PD with depression [71.1 ± 9.9 years, 12F, 2M]	1.5 T	14 manual ROIs	FA: PD with depression < PD without depression only in frontal white matter (anterior cingulum); fornix not evaluated but no group difference in temporal white matter.
	14 PD without depression [69.3 ± 8.1 years, 10F, 4M]	6 directions		
		4 mm slice		
Kim et al., 2013	64 PD [63.0 ± 8.9 years, 22M, 42F]	3 T	TBSS	MD: PD > HC
	64 HC [62.9 ± 9.0 years, 22M, 44F]	15 directions		
		2 mm slice		
Zheng et al., 2014	16 PD [62.2 ± 9.6 years, 11M, 5F]	3 T	40 ROIs	↑ MD = ↓ Non-verbal memory scores (short-term)
		20 directions		
		2 mm slice		
**EPILEPSY**				
Liu et al., 2011a	15 JME patients [21 ± 4 (17–32 years), 3M, 12F] vs. 15 HC [21 ± 4 (17–31 years), 3M, 12F]	1.5 T	Tractography	FA: JME < HC
	17 IGE-GTC [21 ± 4 (18–31 years), 7M, 3F] vs. 10 HC [21 ± 4 (18–30 years), 7M, 3F]	6 directions		FA: IGE-GTC = HC
		1.5 mm slice		
Kuzniecky et al., 1999	35 MTS suspected (age, sex not reported)	1.5 T	Manual ROIs	Asymmetric size
	50 MTLE [32 (17–42 years), 19M, 31F]	MRI		86% of MTLE patients had atrophy ipsilateral to hippocampal atrophy
	17 HC [35 (24–41 years), 8M, 9F]	1.5 mm slice, no gap		
Ozturk et al., 2008	35 MTS suspected (age, sex not reported)	3 T	Visual evaluation (Blinded to Grouping)	Asymmetric size
	353 HC [49.2 (7–87 years), 134M, 219F]	MTR		MTS: 34.3% (12/35)
		1.5 mm slice		HC: 7.9% (28/353)
Kim et al., 1995	33 preHS [31.5 (13–57 years), 19M, 14F]	1.5 T	Visual evaluation (Blinded to Grouping)	Asymmetric size
	7 postHS [27 917–40 years), 3M, 4F]	MRI		preHS: 42%
	34 HC [33.8 (14–56 years), 17M, 17F]	3 mm slice		postHS: 74%
				HC: 6%
**TRAUMATIC BRAIN INJURY (TBI)**				
Gale et al., 1993	27 TBI	MRI	Fornix-to-brain ratios (FBR)	FBR: TBI < HC
	18 HC			Atrophy in TBI
	Only women			No correlation between FBR and neuropsychological outcome.
Tate and Bigler, 2000	86 TBI [30 ± 11.73 (16–65 years), 58M, 28F]	1.5 T	ROI	Area: TBI< HC
	46 HC [37.21 ± 13.08 (16–65 years), 31M, 15F]	MRI		In TBI: ↓Area_fornix α ↓vol_hippocampus α ↑ injury severity
		5 mm slice, 2 mm gap		No correlation in HC
				No correlation between area and memory performance (GMI and WMS-R)
Tomaiuolo et al., 2004	19 TBI [35.5 ± 14.71 (17–68 years), 12M, 7F]	1.5 T	ROI	Volume: TBI< HC
	19 HC [37.4 ± 15.18 (18–72 years), 12M, 7F]	MRI		↓volume α ↓memory performance [Immediate and delayed recall of both RCFT and WMS (word list)]
		1 mm slice		
Kinnunen et al., 2011	28 TBI [38.9 ± 12.2 years, 21M, 7F]	3 T	TBSS	In TBI and HC, ↓ FA α ↓associative memory and learning performance (Immediate recall DPT)
	26 HC [35.4 ± 11.1 years, 12M, 14F]	16 directions		
		2 mm slice		
Palacios et al., 2011	15 TBI [23.6 ± 4.79 (18–32 years), 11M, 4F]	1.5 T	TBSS	FA: TBI < HC
	16 HC [23.7 ± 4.8 (18–32 years), 9M, 7F]	25 directions	ROI	In TBI: ↓FA in fornix α with worse declarative memory but not with working memory; ↓FA in SLF α with working memory
		5 mm slice		
Adnan et al., 2013	29 TBI [5 and 30 months post-injury]		ROI	FA: TBI < HC

α: correlate.

MRI, Magnetic Resonance Imaging; T, Tesla; MTR, magnetization transfer ratio; TBSS, Tract-based spatial statistic; VBA, Voxel-based analysis; ROI, Region of Interest; MO, mode of anisotropy; FA, fractional anisotropy; MD, Mean Diffusivity, AxD, Axial diffusivity; RD, Radial diffusivity.

MS, Multiple sclerosis; PD, Parkinson's disease; RRMS, relapsing-remitting multiple sclerosis; SPMS, secondary progressive multiple sclerosis; PPMS, primary progressive multiple sclerosis; CISSMS, clinically isolated syndrome suggestive of multiple sclerosis; JME, juvenile myoclonic epilepsy; IGE-GTC, generalized tonic–clonic seizures; MTLE, Mesial temporal lobe epilepsy; MS, Mesial Temporal Sclerosis; preHS, pre-surgical hippocampal sclerosis; postHS, post-surgical hippocampal sclerosis; TBI,Traumatic brain injury.

EDSS, Expanded Disability Status Scale; 9-HPT, 9-Hole Peg Test; PASAT-3, Paced Auditory serial Addition Task-3, second version; GMI, General Memory Index score; WMS–R, Wechsler Memory Scale–Revised; RCFT, Rey Complex Fig Test; BVMT-R, Brief Visuospatial Memory Test-Revised; SDMT, Symbol Digit Modalities Test.

Altogether, these findings showed that DTI metrics in the fornix are consistently abnormal in MS patients. Since DTI measures in the fornix can assess disease severity, they may be useful for monitoring MS disease progression. Furthermore, forniceal DTI metrics correlated with hippocampal volume in patients with MS, and DTI measures in the fornix had an even stronger association with visual and episodic memory than the hippocampal volume (Koenig et al., 2014). Therefore, similar to patients who convert from MCI to AD, longitudinal forniceal DTI measures may be useful in predicting hippocampal abnormalities and memory deficits in MS patients.

***Parkinson's disease.*** Parkinson's disease (PD) is most commonly regarded as a movement disorder (Gelb et al., 1999), since degeneration of the nigrostriatal dopaminergic system leads to dysfunction of the motor system with the four cardinal signs of tremors, bradykinesia, rigidity and postural instability. However, dopamine also mediates attention and working memory, which are requried for most higher level cognitive function, Therefore, PD patients commonly develop dementia and cognitive deficits including deficits in executive function, attention, language and memory (Zgaljardic et al., 2003; McKinlay et al., 2010). Few studies investigated the fornix in PD patients using DTI. Similar to AD patients, MD of the fornix was higher in PD patients than in healthy adults (Kim et al., 2013), and higher MD correlated with worse short-term non-verbal memory (Zheng et al., 2014). However, since many dementia patients have co-occurrence of AD and PD, it remains unclear whether the fornix abnormalities are related specifically to PD. Depression is also common amongst PD patients, and those with depression showed lower FA in the frontal white matter than PD patients without depression; although the fornix was not specifically evaluated in this study, and the temporal white matter showed no group difference (Matsui et al., 2007). Another DTI study found that PD patients with excessive daytime sleepiness (Epsworth Sleepiness Scale ≥ 10) had significantly lower FA in their fornix compared to controls (Matsui et al., 2006). Therefore, abnormalities in the fornix appear to contribute to the co-morbid symptoms beyond the extrapyramidal system, such as memory deficits and excessive daytime sleepiness in patients with PD.

***Epilepsy.*** Since a large number of etiologies exist for epilepsy, the fornix may or may not be affected depending on whether this major efferent white matter tract from the hippocampi is affected by the lesion or condition that caused the epilepsy. For instance, mesial temporal sclerosis (MTS) causes temporal lobe epilepsy (TLE), and is frequently accompanied by forniceal atrophy and lower FA when the fornix of these patients are compared to non-epileptic controls (Baldwin et al., 1994; Kim et al., 1995). Decreased fornix volumes and lower FA were often associated with ipsilateral hippocampal sclerosis, both quantitatively and qualitatively (Baldwin et al., 1994; Kuzniecky et al., 1999), and appear to be a good predictor of TLE with accurate lateralization. Therefore, evaluating the fornix and its asymmetry, even with visual interpretations, may be useful in support of presurgical planning (i.e., for surgical resection) for patients with medically intractable TLE. In addition, low frequency depth electrode stimulation of the fornix led to hippocampal and posterior cingulum responses, demonstrating these functional connections, and reduced interictal epileptiform discharges and seizures in patients with intractable mesial temporal lobe epilepsy, without affecting their memory (Koubeissi et al., 2013). Depth electrode stimulation of the fornix also led to either ipsilateral or contralateral hippocampal responses, which again confirmed these neural pathway connections and explained how seizure discharge might spread between homotopic mesial temporal structures without neocortical involvement (Lacuey et al., 2014).

Lastly, a recent DTI study, using tractography, found that patients with juvenile myoclonic epilepsy (JME) had lower FA in the crus of the fornix, body of the corpus callosum and many other major white matter tracts, but not in those with only generalized tonic-clonic seizures, suggesting different neuroanatomical substrates in these two different types of idiopathic generalized epilepsies (Liu et al., 2011a). Taken together, these studies demonstrate that the fornix may play a role in mediating seizure spreads across the cerebral hemispheres both in patients with temporal lobe epilepsy as well as in generalized epilepsies. However, it may also be a treatment target for deep brain stimulation or surgical approaches in these patients.

***Traumatic Brain Injury (TBI)*** results from physical forces that damage the brain, which may cause cognitive impairments such as memory and attention deficits. TBI is also associated with atrophy of the fornix (Gale et al., 1993; Tate and Bigler, 2000; Tomaiuolo et al., 2004). The fornix is particularly susceptible to physical shearing forces (Tate and Bigler, 2000), probably due to its delicate fiber tracts that straddle both cerebral hemispheres. The effects of TBI on the fornix volume have been examined in relation to memory. However, only one of the three studies (Tate and Bigler, 2000; Gale et al., 1993; Tomaiuolo et al., 2004) found a correlation between forniceal atrophy and memory performance (Tomaiuolo et al., 2004). However, using DTI with TBSS, white matter abnormalities were observed in several regions of the brain in TBI patients, but only lower FA in the fornix correlated with worse performance in associative memory and learning in both the TBI and healthy control groups (Kinnunen et al., 2011). In another study, patients with diffuse TBI, which leads to diffuse axonal injury, had globally decreased FA in the brain. However, regional analyses showed that lower FA in the superior longitudinal fasciculus was associated with working memory deficits, while lower forniceal FA was associated with poorer declarative memory in these TBI patients with diffuse injuries (Palacios et al., 2011). Lower forniceal FA and memory deficits were consistently found in TBI patients compared to healthy controls (Palacios et al., 2011; Adnan et al., 2013), suggesting a critical role of the fornix integrity in the development of memory impairments after TBI.

## Discussion

### Limitations and future studies

Several factors have limited the study of the fornix. First, the anatomy of the fornix makes it difficult to evaluate the abnormalities in this brain structure. Specific regions of the fornix (i.e., column, crus or pre-commissural fornix) are even more difficult to visualize or quantify. DTI has improved the visualization of the fornix, which has led to many more studies of this structure in several neurological disorders. However, the forniceal DTI measures in the published studies are often affected by the fornix's close proximity to the ventricles, which can lead to partial volume effects from the CSF in the ventricles. While CSF suppression (using inversion recovery pulses on MRI) would suppress the signals from CSF, most of the DTI studies did not apply such CSF signal suppression during the image acquisition. Partial volume effect from adjacent CSF signal may generate biased (higher) diffusivity and (lower) FA values of the fornix although this structure is generally well delineated on DTI. Nevertheless, refined methods have been developed to minimize the CSF partial volume effect on DTI and obtain higher resolution images. These improved DTI acquisition methods include reducing the repetition time and using non-zero minimum diffusion weighting (Baron and Beaulieu, 2014), or increased the image resolution of DTI (Herbst et al., 2014) by combining multiplexed sensitivity encoding (Chen et al., 2013) and prospective motion correction (Zaitsev et al., 2006; Herbst et al., 2012; Gumus et al., 2014). Others have developed novel criteria for DTI metric selection (Pasternak et al., 2010) using relationships between distribution and distance of the measured diffusion quantities or the use of multi-contrast MRI (Tang et al., 2014) with an automated parcellation atlas, which may further delineate and accurately assess how the fornix might be affected in various brain disorders. Second, the various methods (e.g., manual or automated ROIs, TBSS, tractography) used to measure the diffusivities and FA in the fornix do not always yield the same results. Systematic comparisons or the use of more than one approach to measure the fornix in the same datasets could provide validation to the abnormal findings in the various brain disorders. Third, the majority of the studies reviewed utilized a cross-sectional design, longitudinal follow-up studies would minimize the potential confounding effects of inter-subject variability (e.g., due to differences in disease severity or illness duration) or premorbid group differences. Intra-subject measurements in longitudinal evaluations are more sensitive in detecting, predicting and monitoring neurodegeneration compared to cross-sectional measures. Despite these limitations, the studies reviewed consistently showed correlations between DTI metrics in the fornix and memory performance of typically aging individuals and in patients with various neurodegenerative and neuropsychiatric disorders. These findings strengthen the role of the fornix as a useful imaging marker to predict memory deficits or impairments.

## Conclusion

The fornix is clearly a critical component of the limbic system and is closely linked to memory performance. Alterations of the fornix are related to cognitive functions in childhood and in later life. In addition, forniceal changes were found in schizophrenia and other psychiatric disorders. Therefore, the fornix appears to be more than a clinical surrogate marker of memory impairments for neurodegenerative and neuroinflammatory diseases, such as Alzheimer's disease and multiple sclerosis. Although the fornix is one of the less heritable brain structures (Jahanshad et al., 2013), few studies reported how genes might influence the typical development or aging of the fornix. Imaging genetics might be useful to further elucidate the role of the fornix in various brain disorders as well as during healthy neurodevelopment and brain aging.

### Conflict of interest statement

The authors declare that the research was conducted in the absence of any commercial or financial relationships that could be construed as a potential conflict of interest.

## References

[B1] Abdul-Rahman M. F. Qiu A. Sim K. (2011). Regionally specific white matter disruptions of fornix and cingulum in schizophrenia. PLoS ONE 6:e18652. 10.1371/journal.pone.0018652 21533181 PMC3077390

[B2] Adnan A. Crawley A. Mikulis D. Moscovitch M. Colella B. Green R. (2013). Moderate-severe traumatic brain injury causes delayed loss of white matter integrity: evidence of fornix deterioration in the chronic stage of injury. Brain Inj. 27, 1415–1422. 10.3109/02699052.2013.823659 24102365

[B3] Aggleton J. P. Vann S. D. Oswald C. J. Good M. (2000). Identifying cortical inputs to the rat hippocampus that subserve allocentric spatial processes: a simple problem with a complex answer. Hippocampus 10, 466–474. 10.1002/1098-1063(2000)10:4<466::AID-HIPO13>3.0.CO;2-Y 10985286

[B4] Arnold S. E. Franz B. R. Gur R. C. Gur R. E. Shapiro R. M. Moberg P. J.. (1995). Smaller neuron size in schizophrenia in hippocampal subfields that mediate cortical-hippocampal interactions. Am. J. Psychiatry 152, 738–748. 7726314 10.1176/ajp.152.5.738

[B5] Asato M. R. Terwilliger R. Woo J. Luna B. (2010). White matter development in adolescence: a DTI study. Cereb. Cortex 20, 2122–2131. 10.1093/cercor/bhp282 20051363 PMC2923214

[B6] Baldwin G. N. Tsuruda J. S. Maravilla K. R. Hamill G. S. Hayes C. E. (1994). The fornix in patients with seizures caused by unilateral hippocampal sclerosis: detection of unilateral volume loss on MR images. AJR Am. J. Roentgenol. 162, 1185–1189. 10.2214/ajr.162.5.8166008 8166008

[B7] Baron C. A. Beaulieu C. (2014). Acquisition strategy to reduce cerebrospinal fluid partial volume effects for improved DTI tractography. Magn. Reson. Med. [Epub ahead of print]. 10.1002/mrm.25226 24723303

[B8] Bossy-Wetzel E. Schwarzenbacher R. Lipton S. A. (2004). Molecular pathways to neurodegeneration. Nat. Med. 10, S2–S9. 10.1038/nm1067 15272266

[B9] Brisch R. Bernstein H. G. Stauch R. Dobrowolny H. Krell D. Truebner K.. (2008). The volumes of the fornix in schizophrenia and affective disorders: a post-mortem study. Psychiatry Res. 164, 265–273. 10.1016/j.pscychresns.2007.12.007 19022630

[B10] Brissart H. Daniel F. Morele E. Leroy M. Debouverie M. Defer G. L. (2011). [Cognitive rehabilitation in multiple sclerosis: a review of the literature]. Rev. Neurol. (Paris) 167, 280–290. 10.1016/j.neurol.2010.07.039 21176930

[B11] Broca P. P. (1890). Anatomie comparée des circonvolutions cérébrales, in Extrait de la “Revue d'Anthropologie,” ed Masson (Paris: Elsevier-France), Sér.2,. T. 1, 385–498.

[B12] Burzynska A. Z. Preuschhof C. Backman L. Nyberg L. Li S. C. Lindenberger U.. (2010). Age-related differences in white matter microstructure: region-specific patterns of diffusivity. Neuroimage 49, 2104–2112. 10.1016/j.neuroimage.2009.09.041 19782758

[B13] Callen D. J. Black S. E. Gao F. Caldwell C. B. Szalai J. P. (2001). Beyond the hippocampus: MRI volumetry confirms widespread limbic atrophy in AD. Neurology 57, 1669–1674. 10.1212/WNL.57.9.1669 11706109

[B14] Canu E. Agosta F. Spinelli E. G. Magnani G. Marcone A. Scola E.. (2013). White matter microstructural damage in Alzheimer's disease at different ages of onset. Neurobiol. Aging 34, 2331–2340. 10.1016/j.neurobiolaging.2013.03.026 23623599

[B15] Chance S. A. Esiri M. M. Crow T. J. (2005). Macroscopic brain asymmetry is changed along the antero-posterior axis in schizophrenia. Schizophr. Res. 74, 163–170. 10.1016/j.schres.2004.09.001 15721996

[B16] Chance S. A. Highley J. R. Esiri M. M. Crow T. J. (1999). Fiber content of the fornix in schizophrenia: lack of evidence for a primary limbic encephalopathy. Am. J. Psychiatry 156, 1720–1724. 10553734 10.1176/ajp.156.11.1720

[B17] Chang L. Friedman J. Ernst T. Zhong K. Tsopelas N. D. Davis K. (2007). Brain metabolite abnormalities in the white matter of elderly schizophrenic subjects: implication for glial dysfunction. Biol. Psychiatry 62, 1396–1404. 10.1016/j.biopsych.2007.05.025 17693392 PMC2222890

[B18] Chen N. K. Guidon A. Chang H. C. Song A. W. (2013). A robust multi-shot scan strategy for high-resolution diffusion weighted MRI enabled by multiplexed sensitivity-encoding (MUSE). Neuroimage 72, 41–7. 10.1016/j.neuroimage.2013.01.038 23370063 PMC3602151

[B19] Copenhaver B. R. Rabin L. A. Saykin A. J. Roth R. M. Wishart H. A. Flashman L. A.. (2006). The fornix and mammillary bodies in older adults with Alzheimer's disease, mild cognitive impairment, and cognitive complaints: a volumetric MRI study. Psychiatry Res. 147, 93–103. 10.1016/j.pscychresns.2006.01.015 16920336

[B20] Crow T. J. Colter N. Frith C. D. Johnstone E. C. Owens D. G. (1989). Developmental arrest of cerebral asymmetries in early onset schizophrenia. Psychiatry Res. 29, 247–253. 2608767 10.1016/0165-1781(89)90053-x

[B21] Cui Y. Sachdev P. S. Lipnicki D. M. Jin J. S. Luo S. Zhu W.. (2012). Predicting the development of mild cognitive impairment: a new use of pattern recognition. Neuroimage 60, 894–901. 10.1016/j.neuroimage.2012.01.084 22289804

[B22] Davenport N. D. Karatekin C. White T. Lim K. O. (2010). Differential fractional anisotropy abnormalities in adolescents with ADHD or schizophrenia. Psychiatry Res. 181, 193–198. 10.1016/j.pscychresns.2009.10.012 20153608 PMC2867087

[B23] Davies D. C. Wardell A. M. Woolsey R. James A. C. (2001). Enlargement of the fornix in early-onset schizophrenia: a quantitative MRI study. Neurosci. Lett. 301, 163–166. 10.1016/S0304-3940(01)01637-8 11257423

[B24] DeLisi L. E. Sakuma M. Kushner M. Finer D. L. Hoff A. L. Crow T. J. (1997). Anomalous cerebral asymmetry and language processing in schizophrenia. Schizophr. Bull. 23, 255–271. 9165636 10.1093/schbul/23.2.255

[B25] Dineen R. A. Bradshaw C. M. Constantinescu C. S. Auer D. P. (2012). Extra-hippocampal subcortical limbic involvement predicts episodic recall performance in multiple sclerosis. PLoS ONE 7:e44942. 10.1371/journal.pone.0044942 23056187 PMC3466267

[B26] Dineen R. A. Vilisaar J. Hlinka J. Bradshaw C. M. Morgan P. S. Constantinescu C. S.. (2009). Disconnection as a mechanism for cognitive dysfunction in multiple sclerosis. Brain 132(Pt 1), 239–249. 10.1093/brain/awn275 18953055

[B27] Douaud G. Menke R. A. Gass A. Monsch A. U. Rao A. Whitcher B.. (2013). Brain microstructure reveals early abnormalities more than two years prior to clinical progression from mild cognitive impairment to Alzheimer's disease. J. Neurosci. 33, 2147–2155. 10.1523/JNEUROSCI.4437-12.2013 23365250 PMC6571077

[B28] Douet V. Chang L. Pritchett A. Lee K. Keating B. Bartsch H.. (2014). Schizophrenia-risk variant rs6994992 in the neuregulin-1 gene on brain developmental trajectories in typically-developing children. Transl. Psychiatry 10.1038/tp.2014.41 24865593 PMC4035723

[B29] Dubois J. Dehaene-Lambertz G. Soares C. Cointepas Y. Le Bihan D. Hertz-Pannier L. (2008). Microstructural correlates of infant functional development: example of the visual pathways. J. Neurosci. 28, 1943–1948. 10.1523/JNEUROSCI.5145-07.2008 18287510 PMC6671431

[B30] Fink F. Eling P. Rischkau E. Beyer N. Tomandl B. Klein J.. (2010). The association between California verbal learning test performance and fibre impairment in multiple sclerosis: evidence from diffusion tensor imaging. Mult. Scler. 16, 332–341. 10.1177/1352458509356367 20150400

[B31] Fitzsimmons J. Hamoda H. M. Swisher T. Terry D. Rosenberger G. Seidman L. J.. (2014). Diffusion tensor imaging study of the fornix in first episode schizophrenia and in healthy controls. Schizophr. Res. 156, 157–160. 10.1016/j.schres.2014.04.022 24837684 PMC4080801

[B32] Fitzsimmons J. Kubicki M. Smith K. Bushell G. Estepar R. S. Westin C. F.. (2009). Diffusion tractography of the fornix in schizophrenia. Schizophr. Res. 107, 39–46. 10.1016/j.schres.2008.10.022 19046624 PMC2646850

[B33] Fletcher E. Raman M. Huebner P. Liu A. Mungas D. Carmichael O.. (2013). Loss of fornix white matter volume as a predictor of cognitive impairment in cognitively normal elderly individuals. JAMA Neurol. 70, 1389–1395. 10.1001/jamaneurol.2013.3263 24018960 PMC4059679

[B34] Gaffan E. A. Gaffan D. Hodges J. R. (1991). Amnesia following damage to the left fornix and to other sites. A comparative study. Brain 114(Pt 3), 1297–1313. 2065251 10.1093/brain/114.3.1297

[B35] Gale S. D. Burr R. B. Bigler E. D. Blatter D. (1993). Fornix degeneration and memory in traumatic brain injury. Brain Res. Bull. 32, 345–349. 8221124 10.1016/0361-9230(93)90198-k

[B36] Garcia-Bengochea F. Friedman W. A. (1987). Persistent memory loss following section of the anterior fornix in humans. A historical review. Surg. Neurol. 27, 361–364. 3103247 10.1016/0090-3019(87)90012-7

[B37] Gelb D. J. Oliver E. Gilman S. (1999). Diagnostic criteria for Parkinson disease. Arch. Neurol. 56, 33–39. 9923759 10.1001/archneur.56.1.33

[B38] Giorgio A. Santelli L. Tomassini V. Bosnell R. Smith S. De Stefano N.. (2010). Age-related changes in grey and white matter structure throughout adulthood. Neuroimage 51, 943–951. 10.1016/j.neuroimage.2010.03.004 20211265 PMC2896477

[B39] Gumus K. Keating B. Poser B. A. Armstrong B. Chang L. Maclaren J.. (2014). Prevention of motion-induced signal loss in diffusion-weighted echo-planar imaging by dynamic restoration of gradient moments. Magn. Reson. Med. 71, 2006–2013. 10.1002/mrm.24857 23821373 PMC4420624

[B40] Hattori T. Sato R. Aoki S. Yuasa T. Mizusawa H. (2012). Different patterns of fornix damage in idiopathic normal pressure hydrocephalus and Alzheimer disease. AJNR Am. J. Neuroradiol. 33, 274–279. 10.3174/ajnr.A2780 22081679 PMC7964782

[B41] Heckers S. Heinsen H. Geiger B. Beckmann H. (1991). Hippocampal neuron number in schizophrenia. A stereological study. Arch. Gen. Psychiatry 48, 1002–1008. 1747014 10.1001/archpsyc.1991.01810350042006

[B42] Herbst M. Maclaren J. Weigel M. Korvink J. Hennig J. Zaitsev M. (2012). Prospective motion correction with continuous gradient updates in diffusion weighted imaging. Magn. Reson. Med. 67, 326–338. 10.1002/mrm.23230 22161984

[B43] Herbst M. Zahneisen B. Knowes B. Zaitsev M. Ernst T. (2014). Prospective motion correction of segmented diffusion weighted EPI. Mag. Reson. Med. [Epub ahead of print]. 10.1002/mrm.25547 25446934 PMC4451442

[B44] Hermoye L. Saint-Martin C. Cosnard G. Lee S. K. Kim J. Nassogne M. C.. (2006). Pediatric diffusion tensor imaging: normal database and observation of the white matter maturation in early childhood. Neuroimage 29, 493–504. 10.1016/j.neuroimage.2005.08.017 16194615

[B45] Hori A. (1995). Unilateral volume loss of the fornix in patients with seizures caused by ipsilateral hippocampal sclerosis. AJR Am. J. Roentgenol. 164, 1304. 10.2214/ajr.164.5.7717266 7717266

[B46] Huang H. Fan X. Weiner M. Martin-Cook K. Xiao G. Davis J.. (2012). Distinctive disruption patterns of white matter tracts in Alzheimer's disease with full diffusion tensor characterization. Neurobiol. Aging 33, 2029–2045. 10.1016/j.neurobiolaging.2011.06.027 21872362 PMC3227739

[B47] Huang H. Xue R. Zhang J. Ren T. Richards L. J. Yarowsky P.. (2009). Anatomical characterization of human fetal brain development with diffusion tensor magnetic resonance imaging. J. Neurosci. 29, 4263–4273. 10.1523/JNEUROSCI.2769-08.2009 19339620 PMC2721010

[B48] Huang H. Zhang J. Wakana S. Zhang W. Ren T. Richards L. J.. (2006). White and gray matter development in human fetal, newborn and pediatric brains. Neuroimage 33, 27–38. 10.1016/j.neuroimage.2006.06.009 16905335

[B49] Jahanshad N. Kochunov P. V. Sprooten E. Mandl R. C. Nichols T. E. Almasy L.. (2013). Multi-site genetic analysis of diffusion images and voxelwise heritability analysis: a pilot project of the ENIGMA-DTI working group. Neuroimage 81, 455–469. 10.1016/j.neuroimage.2013.04.061 23629049 PMC3729717

[B50] Jang S. H. Cho S. H. Chang M. C. (2011). Age-related degeneration of the fornix in the human brain: a diffusion tensor imaging study. Int. J. Neurosci. 121, 94–100. 10.3109/00207454.2010.531894 21062216

[B51] Kantarci K. Senjem M. L. Avula R. Zhang B. Samikoglu A. R. Weigand S. D.. (2011). Diffusion tensor imaging and cognitive function in older adults with no dementia. Neurology 77, 26–34. 10.1212/WNL.0b013e31822313dc 21593440 PMC3127333

[B52] Kasai K. Shenton M. E. Salisbury D. F. Hirayasu Y. Lee C. U. Ciszewski A. A.. (2003). Progressive decrease of left superior temporal gyrus gray matter volume in patients with first-episode schizophrenia. Am. J. Psychiatry 160, 156–164. 10.1176/appi.ajp.160.1.156 12505815 PMC2845847

[B53] Kendi M. Kendi A. T. Lehericy S. Ducros M. Lim K. O. Ugurbil K.. (2008). Structural and diffusion tensor imaging of the fornix in childhood- and adolescent-onset schizophrenia. J. Am. Acad. Child Adolesc. Psychiatry 47, 826–832. 10.1097/CHI.Ob013e318172ef36 18520955

[B54] Kern K. C. Ekstrom A. D. Suthana N. A. Giesser B. S. Montag M. Arshanapalli A.. (2012). Fornix damage limits verbal memory functional compensation in multiple sclerosis. Neuroimage 59, 2932–2940. 10.1016/j.neuroimage.2011.09.071 22001266

[B55] Kim H. J. Kim S. J. Kim H. S. Choi C. G. Kim N. Han S.. (2013). Alterations of mean diffusivity in brain white matter and deep gray matter in Parkinson's disease. Neurosci. Lett. 550, 64–68. 10.1016/j.neulet.2013.06.050 23831353

[B56] Kim J. H. Tien R. D. Felsberg G. J. Osumi A. K. Lee N. (1995). Clinical significance of asymmetry of the fornix and mamillary body on MR in hippocampal sclerosis. AJNR Am. J. Neuroradiol. 16, 509–515. 7793375 PMC8337654

[B57] King E. C. Pattwell S. S. Sun A. Glatt C. E. Lee F. S. (2013). Nonlinear developmental trajectory of fear learning and memory. Ann. N.Y. Acad. Sci. 1304, 62–69. 10.1111/nyas.12280 24176014 PMC4155981

[B58] Kinnunen K. M. Greenwood R. Powell J. H. Leech R. Hawkins P. C. Bonnelle V.. (2011). White matter damage and cognitive impairment after traumatic brain injury. Brain 134(Pt 2), 449–463. 10.1093/brain/awq347 21193486 PMC3030764

[B59] Kochunov P. Hong L. E. (2014). Neurodevelopmental and neurodegenerative models of schizophrenia: white matter at the center stage. Schizophr. Bull. 40, 721–728. 10.1093/schbul/sbu070 24870447 PMC4059450

[B60] Koenig K. A. Lowe M. J. Lin J. Sakaie K. E. Stone L. Bermel R. A.. (2013). Sex differences in resting-state functional connectivity in multiple sclerosis. AJNR Am. J. Neuroradiol. 34, 2304–2311. 10.3174/ajnr.A3630 23811974 PMC7965207

[B61] Koenig K. A. Sakaie K. E. Lowe M. J. Lin J. Stone L. Bermel R. A.. (2014). Hippocampal volume is related to cognitive decline and fornicial diffusion measures in multiple sclerosis. Magn. Reson. Imaging 32, 354–358. 10.1016/j.mri.2013.12.012 24512796 PMC4025957

[B62] Koubeissi M. Z. Kahriman E. Syed T. U. Miller J. Durand D. M. (2013). Low-frequency electrical stimulation of a fiber tract in temporal lobe epilepsy. Ann. Neurol. 74, 223–231. 10.1002/ana.23915 23613463

[B63] Kuroki N. Kubicki M. Nestor P. G. Salisbury D. F. Park H. J. Levitt J. J.. (2006). Fornix integrity and hippocampal volume in male schizophrenic patients. Biol. Psychiatry 60, 22–31. 10.1016/j.biopsych.2005.09.021 16406249 PMC2768597

[B64] Kuzniecky R. Bilir E. Gilliam F. Faught E. Martin R. Hugg J. (1999). Quantitative MRI in temporal lobe epilepsy: evidence for fornix atrophy. Neurology 53, 496–501. 10449110 10.1212/wnl.53.3.496

[B65] Lacuey N. Zonjy B. Kahriman E. S. Kaffashi F. Miller J. Luders H. O. (2014). Functional connectivity between right and left mesial temporal structures. Brain Struct. Funct. [Epub ahead of print]. 10.1007/s00429-014-0810-0 24908158

[B66] Lebel C. Gee M. Camicioli R. Wieler M. Martin W. Beaulieu C. (2012). Diffusion tensor imaging of white matter tract evolution over the lifespan. Neuroimage 60, 340–352. 10.1016/j.neuroimage.2011.11.094 22178809

[B67] Lee C. E. Danielian L. E. Thomasson D. Baker E. H. (2009). Normal regional fractional anisotropy and apparent diffusion coefficient of the brain measured on a 3 T MR scanner. Neuroradiology 51, 3–9. 10.1007/s00234-008-0441-3 18704391

[B68] Lee S. H. Kubicki M. Asami T. Seidman L. J. Goldstein J. M. Mesholam-Gately R. I.. (2013). Extensive white matter abnormalities in patients with first-episode schizophrenia: a diffusion tensor iimaging (DTI) study. Schizophr. Res. 143, 231–238. 10.1016/j.schres.2012.11.029 23290268 PMC4354799

[B69] Liu M. Concha L. Beaulieu C. Gross D. W. (2011a). Distinct white matter abnormalities in different idiopathic generalized epilepsy syndromes. Epilepsia 52, 2267–2275. 10.1111/j.1528-1167.2011.03313.x 22092238

[B70] Liu Y. Spulber G. Lehtimäki K. K. Könönen M. Hallikainen I. Gröhn H.. (2011b). Diffusion tensor imaging and tract-based spatial statistics in Alzheimer's disease and mild cognitive impairment. Neurobiol. Aging 32, 1558–1571. 10.1016/j.neurobiolaging.2009.10.006 19913331

[B71] Luck D. Malla A. K. Joober R. Lepage M. (2010). Disrupted integrity of the fornix in first-episode schizophrenia. Schizophr. Res. 119, 61–64. 10.1016/j.schres.2010.03.027 20409692

[B71a] Maier-Hein K. H. Brunner R. Lutz K. Henze R. Parzer P. Feigl N. (2014). Disorder-specific white matter alterations in adolescent borderline personality disorder. [Research Support, Non-U.S. Gov't]. Biol. Psychiatry 75, 81–88 10.1016/j.biopsych.2013.03.031 23768862

[B72] Matsui H. Nishinaka K. Oda M. Niikawa H. Komatsu K. Kubori T.. (2006). Disruptions of the fornix fiber in Parkinsonian patients with excessive daytime sleepiness. Parkinsonism Relat. Disord. 12, 319–322. 10.1016/j.parkreldis.2006.01.007 16621664

[B73] Matsui H. Nishinaka K. Oda M. Niikawa H. Komatsu K. Kubori T.. (2007). Depression in Parkinson's disease. Diffusion tensor imaging study. J. Neurol. 254, 1170–1173. 10.1007/s00415-006-0236-6 17710361

[B74] McDonald B. Highley J. R. Walker M. A. Herron B. M. Cooper S. J. Esiri M. M.. (2000). Anomalous asymmetry of fusiform and parahippocampal gyrus gray matter in schizophrenia: a postmortem study. [Comparative Study Research Support, Non-U.S. Gov't]. Am. J. Psychiatry 157, 40–47. 10.1176/ajp.157.1.40 10618011

[B75] McKinlay A. Grace R. C. Dalrymple-Alford J. C. Roger D. (2010). Characteristics of executive function impairment in Parkinson's disease patients without dementia. J. Int. Neuropsychol. Soc. 16, 268–277. 10.1017/S1355617709991299 20003582

[B76] Metzler-Baddeley C. Hunt S. Jones D. K. Leemans A. Aggleton J. P. O'Sullivan M. J. (2012). Temporal association tracts and the breakdown of episodic memory in mild cognitive impairment. Neurology 79, 2233–2240. 10.1212/WNL.0b013e31827689e8 23175726 PMC3542350

[B77] Metzler-Baddeley C. Jones D. K. Belaroussi B. Aggleton J. P. O'Sullivan M. J. (2011). Frontotemporal connections in episodic memory and aging: a diffusion MRI tractography study. J. Neurosci. 31, 13236–13245. 10.1523/JNEUROSCI.2317-11.2011 21917806 PMC6623273

[B78] Michielse S. Coupland N. Camicioli R. Carter R. Seres P. Sabino J.. (2010). Selective effects of aging on brain white matter microstructure: a diffusion tensor imaging tractography study. Neuroimage 52, 1190–1201. 10.1016/j.neuroimage.2010.05.019 20483378

[B79] Mielke M. M. Kozauer N. A. Chan K. C. George M. Toroney J. Zerrate M.. (2009). Regionally-specific diffusion tensor imaging in mild cognitive impairment and Alzheimer's disease. Neuroimage 46, 47–55. 10.1016/j.neuroimage.2009.01.054 19457371 PMC2688089

[B80] Mielke M. M. Okonkwo O. C. Oishi K. Mori S. Tighe S. Miller M. I.. (2012). Fornix integrity and hippocampal volume predict memory decline and progression to Alzheimer's disease. Alzheimers Dement. 8, 105–113. 10.1016/j.jalz.2011.05.2416 22404852 PMC3305232

[B81] Mielke M. M. Vemuri P. Rocca W. A. (2014). Clinical epidemiology of Alzheimer's disease: assessing sex and gender differences. Clin. Epidemiol. 6, 37–48. 10.2147/CLEP.S37929 24470773 PMC3891487

[B82] Mitchell R. L. Crow T. J. (2005). Right hemisphere language functions and schizophrenia: the forgotten hemisphere? Brain 128(Pt 5), 963–978. 10.1093/brain/awh466 15743870

[B83] Mitelman S. A. Shihabuddin L. Brickman A. M. Buchsbaum M. S. (2005). Cortical intercorrelations of temporal area volumes in schizophrenia. Schizophr. Res. 76, 207–229. 10.1016/j.schres.2005.01.010 15949654

[B84] Nestor P. G. Kubicki M. Kuroki N. Gurrera R. J. Niznikiewicz M. Shenton M. E.. (2007). Episodic memory and neuroimaging of hippocampus and fornix in chronic schizophrenia. Psychiatry Res. 155, 21–28. 10.1016/j.pscychresns.2006.12.020 17395435

[B85] Nolte J. (2009). The Human Brain: An Introduction Ot Its Functional Anatomy. Philadelphia, PA: Mosby Elsevier.

[B86] Nowrangi M. A. Lyketsos C. G. Leoutsakos J. M. Oishi K. Albert M. Mori S.. (2013). Longitudinal, region-specific course of diffusion tensor imaging measures in mild cognitive impairment and Alzheimer's disease. Alzheimers Dement. 9, 519–528. 10.1016/j.jalz.2012.05.2186 23245561 PMC3639296

[B87] Oishi K. Mielke M. M. Albert M. Lyketsos C. G. Mori S. (2012). The fornix sign: a potential sign for Alzheimer's disease based on diffusion tensor imaging. J. Neuroimaging 22, 365–374. 10.1111/j.1552-6569.2011.00633.x 21848679 PMC3256282

[B87a] Ozturk A. Yousem D. M. Mahmood A. El Sayed S. (2008). Prevalence of asymmetry of mamillary body and fornix size on MR imaging. AJNR Am. J. Neuroradiol. 29, 384–387. 10.3174/ajnr.A0801 17989375 PMC8119011

[B88] Pagani E. Agosta F. Rocca M. A. Caputo D. Filippi M. (2008). Voxel-based analysis derived from fractional anisotropy images of white matter volume changes with aging. Neuroimage 41, 657–667. 10.1016/j.neuroimage.2008.03.021 18442927

[B89] Palacios E. M. Fernandez-Espejo D. Junque C. Sanchez-Carrion R. Roig T. Tormos J. M.. (2011). Diffusion tensor imaging differences relate to memory deficits in diffuse traumatic brain injury. BMC Neurol. 11:24. 10.1186/1471-2377-11-24 21345223 PMC3050687

[B90] Papez J. W. (1937). A proposed mechanism of emotion. 1937. J. Neuropsychiatry Clin. Neurosci. 7, 103–112. 7711480 10.1176/jnp.7.1.103

[B91] Pasternak O. Sochen N. Basser P. J. (2010). The effect of metric selection on the analysis of diffusion tensor MRI data. Neuroimage 49, 2190–2204. 10.1016/j.neuroimage.2009.10.071 19879947 PMC2975386

[B92] Pelletier A. Periot O. Dilharreguy B. Hiba B. Bordessoules M. Peres K.. (2013). Structural hippocampal network alterations during healthy aging: a multi-modal MRI study. Front. Aging Neurosci. 5:84. 10.3389/fnagi.2013.00084 24367331 PMC3852215

[B93] Penfield W. Milner B. (1958). Memory deficit produced by bilateral lesions in the hippocampal zone. AMA Arch. Neurol. Psychiatry 79, 475–497. 13519951 10.1001/archneurpsyc.1958.02340050003001

[B94] Peper J. S. Brouwer R. M. Schnack H. G. van Baal G. C. van Leeuwen M. van den Berg S. M.. (2008). Cerebral white matter in early puberty is associated with luteinizing hormone concentrations. Psychoneuroendocrinology 33, 909–915. 10.1016/j.psyneuen.2008.03.017 18640784

[B95] Peper J. S. Brouwer R. M. Schnack H. G. van Baal G. C. van Leeuwen M. van den Berg S. M.. (2009). Sex steroids and brain structure in pubertal boys and girls. Psychoneuroendocrinology 34, 332–342. 10.1016/j.psyneuen.2008.09.012 18980810

[B96] Petersen R. C. (2004). Mild cognitive impairment as a diagnostic entity. J. Intern. Med. 256, 183–194. 10.1111/j.1365-2796.2004.01388.x 15324362

[B97] Petersen R. C. Doody R. Kurz A. Mohs R. C. Morris J. C. Rabins P. V.. (2001). Current concepts in mild cognitive impairment. Arch. Neurol. 58, 1985–1992. 10.1001/archneur.58.12.1985 11735772

[B98] Pihlajamaki M. Jauhiainen A. M. Soininen H. (2009). Structural and functional MRI in mild cognitive impairment. Curr. Alzheimer Res. 6, 179–185. 10.2174/156720509787602898 19355853

[B99] Pivneva T. A. (2008). Microglia in normal condition and pathology. Fiziol. Zh. 54, 81–89. 19058517

[B100] Rados M. Judas M. Kostovic I. (2006). *In vitro* MRI of brain development. Eur. J. Radiol. 57, 187–198. 10.1016/j.ejrad.2005.11.019 16439088

[B101] Rajmohan V. Mohandas E. (2007). The limbic system. Indian J. Psychiatry, 49, 132–139. 10.4103/0019-5545.33264 20711399 PMC2917081

[B102] Ranjeva J. P. Audoin B. Au Duong M. V. Ibarrola D. Confort-Gouny S. Malikova I.. (2005). Local tissue damage assessed with statistical mapping analysis of brain magnetization transfer ratio: relationship with functional status of patients in the earliest stage of multiple sclerosis. AJNR Am. J. Neuroradiol. 26, 119–127. 15661713 PMC7975013

[B103] Rapoport J. L. Giedd J. N. Gogtay N. (2012). Neurodevelopmental model of schizophrenia: update 2012. Mol. Psychiatry 17, 1228–1238. 10.1038/mp.2012.23 22488257 PMC3504171

[B104] Ringman J. M. O'Neill J. Geschwind D. Medina L. Apostolova L. G. Rodriguez Y.. (2007). Diffusion tensor imaging in preclinical and presymptomatic carriers of familial Alzheimer's disease mutations. Brain 130(Pt 7), 1767–1776. 10.1093/brain/awm102 17522104

[B105] Roosendaal S. D. Geurts J. J. Vrenken H. Hulst H. E. Cover K. S. Castelijns J. A.. (2009). Regional DTI differences in multiple sclerosis patients. Neuroimage 44, 1397–1403. 10.1016/j.neuroimage.2008.10.026 19027076

[B106] Rudebeck S. R. Scholz J. Millington R. Rohenkohl G. Johansen-Berg H. Lee A. C. (2009). Fornix microstructure correlates with recollection but not familiarity memory. J. Neurosci. 29, 14987–14992. 10.1523/JNEUROSCI.4707-09.2009 19940194 PMC2825810

[B107] Sala S. Agosta F. Pagani E. Copetti M. Comi G. Filippi M. (2012). Microstructural changes and atrophy in brain white matter tracts with aging. Neurobiol. Aging 33, 488–498. 10.1016/j.neurobiolaging.2010.04.027 20594616

[B108] Samson R. D. Barnes C. A. (2013). Impact of aging brain circuits on cognition. Eur. J. Neurosci. 37, 1903–1915. 10.1111/ejn.12183 23773059 PMC3694726

[B109] Sasson E. Doniger G. M. Pasternak O. Tarrasch R. Assaf Y. (2013). White matter correlates of cognitive domains in normal aging with diffusion tensor imaging. Front. Neurosci. 7:32. 10.3389/fnins.2013.00032 23493587 PMC3595518

[B110] Schaefer J. Giangrande E. Weinberger D. R. Dickinson D. (2013). The global cognitive impairment in schizophrenia: consistent over decades and around the world. Schizophr. Res. 150, 42–50. 10.1016/j.schres.2013.07.009 23911259 PMC4196267

[B111] Schmithorst V. J. Holland S. K. Dardzinski B. J. (2008). Developmental differences in white matter architecture between boys and girls. Hum. Brain Mapp. 29, 696–710. 10.1002/hbm.20431 17598163 PMC2396458

[B112] Sexton C. E. Kalu U. G. Filippini N. Mackay C. E. Ebmeier K. P. (2011). A meta-analysis of diffusion tensor imaging in mild cognitive impairment and Alzheimer's disease. Neurobiol. Aging 32, 2322. 10.1016/j.neurobiolaging.2010.05.019 20619504

[B112a] Sexton C. E. Mackay C. E. Lonie J. A. Bastin M. E. Terriére E. O'Carroll R. E.. (2010). MRI correlates of episodic memory in Alzheimer's disease, mild cognitive impairment, and healthy aging. [Research Support, Non-U.S. Gov't]. Psychiatry Res. 184, 57–62. 10.1016/j.pscychresns.2010.07.005 20832251

[B113] Simmonds D. J. Hallquist M. N. Asato M. Luna B. (2014). Developmental stages and sex differences of white matter and behavioral development through adolescence: a longitudinal diffusion tensor imaging (DTI) study. Neuroimage 92, 356–368. 10.1016/j.neuroimage.2013.12.044 24384150 PMC4301413

[B114] Smith S. M. Jenkinson M. Johansen-Berg H. Rueckert D. Nichols T. E. Mackay C. E.. (2006). Tract-based spatial statistics: voxelwise analysis of multi-subject diffusion data. [Research Support, Non-U.S. Gov't]. Neuroimage 31, 1487–1505. 10.1016/j.neuroimage.2006.02.024 16624579

[B115] Song S. K. Sun S. W. Ju W. K. Lin S. J. Cross A. H. Neufeld A. H. (2003). Diffusion tensor imaging detects and differentiates axon and myelin degeneration in mouse optic nerve after retinal ischemia. Neuroimage 20, 1714–1722. 10.1016/j.neuroimage.2003.07.005 14642481

[B116] Song S. K. Sun S. W. Ramsbottom M. J. Chang C. Russell J. Cross A. H. (2002). Dysmyelination revealed through MRI as increased radial (but unchanged axial) diffusion of water. Neuroimage 17, 1429–1436. 10.1006/nimg.2002.1267 12414282

[B117] Song S. K. Yoshino J. Le T. Q. Lin S. J. Sun S. W. Cross A. H.. (2005). Demyelination increases radial diffusivity in corpus callosum of mouse brain. Neuroimage 26, 132–40. 10.1016/j.neuroimage.2005.01.028 15862213

[B118] Squire L. R. Zola-Morgan S. (1991). The medial temporal lobe memory system. Science 253, 1380–1386. 1896849 10.1126/science.1896849

[B119] Stadlbauer A. Salomonowitz E. Strunk G. Hammen T. Ganslandt O. (2008). Quantitative diffusion tensor fiber tracking of age-related changes in the limbic system. Eur. Radiol. 18, 130–137. 10.1007/s00330-007-0733-8 17701181

[B120] Stricker N. H. Schweinsburg B. C. Delano-Wood L. Wierenga C. E. Bangen K. J. Haaland K. Y.. (2009). Decreased white matter integrity in late-myelinating fiber pathways in Alzheimer's disease supports retrogenesis. Neuroimage 45, 10–16. 10.1016/j.neuroimage.2008.11.027 19100839 PMC2782417

[B121] Sullivan E. V. Rohlfing T. Pfefferbaum A. (2010). Quantitative fiber tracking of lateral and interhemispheric white matter systems in normal aging: relations to timed performance. Neurobiol. Aging 31, 464–481. 10.1016/j.neurobiolaging.2008.04.007 18495300 PMC2815144

[B122] Syc S. B. Harrison D. M. Saidha S. Seigo M. Calabresi P. A. Reich D. S. (2013). Quantitative MRI demonstrates abnormality of the fornix and cingulum in multiple sclerosis. Mult. Scler. Int. 2013:838719. 10.1155/2013/838719 23476776 PMC3586491

[B123] Takei K. Yamasue H. Abe O. Yamada H. Inoue H. Suga M.. (2008). Disrupted integrity of the fornix is associated with impaired memory organization in schizophrenia. Schizophr. Res. 103, 52–61. 10.1016/j.schres.2008.03.008 18442897

[B124] Tang X. Yoshida S. Hsu J. Huisman T. A. Faria A. V. Oishi K.. (2014). Multi-contrast multi-atlas parcellation of diffusion tensor imaging of the human brain. PLoS ONE 9:e96985. 10.1371/journal.pone.0096985 24809486 PMC4014574

[B125] Tate D. F. Bigler E. D. (2000). Fornix and hippocampal atrophy in traumatic brain injury. Learn. Mem. 7, 442–446. 10.1101/lm.33000 11112803

[B126] Teipel S. J. Grothe M. Lista S. Toschi N. Garaci F. G. Hampel H. (2013). Relevance of magnetic resonance imaging for early detection and diagnosis of Alzheimer disease. Med. Clin. North Am. 97, 399–424. 10.1016/j.mcna.2012.12.013 23642578

[B127] Tomaiuolo F. Carlesimo G. A. Di Paola M. Petrides M. Fera F. Bonanni R.. (2004). Gross morphology and morphometric sequelae in the hippocampus, fornix, and corpus callosum of patients with severe non-missile traumatic brain injury without macroscopically detectable lesions: a T1 weighted MRI study. J. Neurol. Neurosurg. Psychiatry 75, 1314–1322. 10.1136/jnnp.2003.017046 15314123 PMC1739237

[B128a] Vernooij M. W. de Groot M. van der Lugt A. Ikram M. A. Krestin G. P. Hofman A.. (2008). White matter atrophy and lesion formation explain the loss of structural integrity of white matter in aging. [Research Support, Non-U.S. Gov't]. Neuroimage 43, 470–477. 10.1016/j.neuroimage.2008.07.052 18755279

[B129] Vita A. De Peri L. Deste G. Sacchetti E. (2012). Progressive loss of cortical gray matter in schizophrenia: a meta-analysis and meta-regression of longitudinal MRI studies. Transl. Psychiatry 2, e190 10.1038/tp.2012.116 23168990 PMC3565772

[B130] Westerhausen R. Kreuder F. Dos Santos Sequeira S. Walter C. Woerner W. Wittling R. A.. (2004). Effects of handedness and gender on macro- and microstructure of the corpus callosum and its subregions: a combined high-resolution and diffusion-tensor MRI study. Brain Res. Cogn. Brain Res. 21, 418–426. 10.1016/j.cogbrainres.2004.07.002 15511657

[B131] White T. Nelson M. Lim K. O. (2008). Diffusion tensor imaging in psychiatric disorders. Top Magn. Reson. Imaging 19 97–109. 10.1097/RMR.0b013e3181809f1e 19363432

[B132] Yanike M. Ferrera V. P. (2014). Representation of outcome risk and action in the anterior caudate nucleus. J. Neurosci. 34, 3279–3290. 10.1523/JNEUROSCI.3818-13.2014 24573287 PMC3935089

[B133] Yasmin H. Aoki S. Abe O. Nakata Y. Hayashi N. Masutani Y.. (2009). Tract-specific analysis of white matter pathways in healthy subjects: a pilot study using diffusion tensor MRI. Neuroradiology 51, 831–840. 10.1007/s00234-009-0580-1 19662389

[B134] Zahajszky J. Dickey C. C. McCarley R. W. Fischer I. A. Nestor P. Kikinis R.. (2001). A quantitative MR measure of the fornix in schizophrenia. Schizophr. Res. 47, 87–97. 10.1016/S0920-9964(00)00051-7 11163548 PMC2845160

[B135] Zahr N. M. Rohlfing T. Pfefferbaum A. Sullivan E. V. (2009). Problem solving, working memory, and motor correlates of association and commissural fiber bundles in normal aging: a quantitative fiber tracking study. Neuroimage 44, 1050–1062. 10.1016/j.neuroimage.2008.09.046 18977450 PMC2632960

[B136] Zaitsev M. Dold C. Sakas G. Hennig J. Speck O. (2006). Magnetic resonance imaging of freely moving objects: prospective real-time motion correction using an external optical motion tracking system. Neuroimage 31, 1038–1050. 10.1016/j.neuroimage.2006.01.039 16600642

[B137] Zgaljardic D. J. Borod J. C. Foldi N. S. Mattis P. (2003). A review of the cognitive and behavioral sequelae of Parkinson's disease: relationship to frontostriatal circuitry. Cogn. Behav. Neurol. 16, 193–210. 10.1097/00146965-200312000-00001 14665819

[B138] Zheng Z. Shemmassian S. Wijekoon C. Kim W. Bookheimer S. Y. Pouratian N. (2014). DTI correlates of distinct cognitive impairments in Parkinson's disease. Hum. Brain Mapp. 35, 1325–1333. 10.1002/hbm.22256 23417856 PMC3664116

[B139] Zhuang L. Sachdev P. S. Trollor J. N. Reppermund S. Kochan N. A. Brodaty H.. (2013). Microstructural white matter changes, not hippocampal atrophy, detect early amnestic mild cognitive impairment. PLoS ONE 8:e58887. 10.1371/journal.pone.0058887 23516569 PMC3597581

[B140] Zhuang L. Wen W. Zhu W. Trollor J. Kochan N. Crawford J.. (2010). White matter integrity in mild cognitive impairment: a tract-based spatial statistics study. Neuroimage 53, 16–25. 10.1016/j.neuroimage.2010.05.068 20595067

